# *Ligusticum sinense* Oliv. cv. Chaxiong Extract Alleviates Nitroglycerin-Induced Migraine-like Responses in Rats: Associations with Glycerophospholipid Metabolism and PI3K/AKT Signaling

**DOI:** 10.3390/ijms27188169

**Published:** 2026-09-14

**Authors:** Yu Zhu, Yanjing Dong, Shiyi Jiang, Qian Qin, Yu He, Danyang Wu, Shouwen Zhang, Juan Wei

**Affiliations:** The Research Center for Traditional Chinese Medicine Resources and Ethnic Minority, Jiangxi University of Chinese Medicine, Nanchang 330004, China; zhuyu2095@jxutcm.edu.cn (Y.Z.); dongyanjing@ncmc.edu.cn (Y.D.); jiangshiyi@jxutcm.edu.cn (S.J.); qinqian@jxutcm.edu.cn (Q.Q.); heyu2@jxutcm.edu.cn (Y.H.); wudanyang@jxutcm.edu.cn (D.W.)

**Keywords:** Chaxiong, migraine, glycerophospholipid metabolism, PI3K/AKT signaling pathway, untargeted metabolomics, network pharmacology

## Abstract

*Ligusticum sinense* Oliv. cv. Chaxiong (Chaxiong) has traditionally been used as a medicinal tea for headache relief in southern China, but its bioactive basis and mechanisms remain unclear. This study integrated ultra-performance liquid chromatography–quadrupole time-of-flight tandem mass spectrometry (UPLC-Q-TOF-MS/MS), untargeted metabolomics, network pharmacology, molecular docking, reverse-transcription quantitative PCR (RT-qPCR), and Western blotting in a nitroglycerin-induced migraine-like rat model. Chaxiong extract alleviated migraine-like behavior, reduced calcitonin gene-related peptide (CGRP), nitric oxide (NO), and tumor necrosis factor-α (TNF-α), increased 5-hydroxytryptamine (5-HT), and ameliorated brain histopathological alterations. A total of 48 constituents and 13 differential serum metabolites were annotated or putatively annotated, respectively. Glycerophospholipid metabolism was the principal metabolic pathway affected by treatment, whereas network pharmacology prioritized phosphatidylinositol 3-kinase/protein kinase B (PI3K/AKT) signaling. Pathway integration identified PIK3CA, PIK3CB, AKT1, AKT2, and INS as candidate bridging targets. Exploratory docking suggested potential interactions between five representative constituents and PIK3CA/AKT1. RT-qPCR showed reduced *Pik3ca* and *Akt1* mRNA expression, while Western blotting showed decreased p-PI3K p85α (Tyr607)/PI3K p85α and p-AKT (Ser473)/AKT1 ratios following treatment. These findings suggest that Chaxiong-associated alleviation of migraine-like responses was accompanied by altered glycerophospholipid metabolism and reduced PI3K/AKT pathway activation.

## 1. Introduction

Migraine is one of the most common primary headache disorders and remains a major cause of disability worldwide, affecting roughly 18% of the global population [1,2]. Recurrent attacks are often accompanied by nausea, photophobia, phonophobia, and significant dysfunction, which impose a heavy burden on patients, families, and healthcare systems [3,4,5]. Although the biological basis of migraine has not been fully clarified, a variety of interrelated mechanisms have been confirmed to be involved in its pathogenesis, including trigeminovascular activation, neurovascular dysfunction, altered neurotransmitter regulation, and the microbiota–gut–brain axis, which has attracted growing research interest in recent years [6]. Current treatments, such as triptans, non-steroidal anti-inflammatory drugs, and CGRP-targeted therapies [7], are effective for some patients. Still, their application is often limited by adverse reactions, high costs, and incomplete efficacy, or focuses solely on symptom relief rather than broader pathophysiological regulation [8]. These limitations continue to draw people’s attention to safer, more functional treatment options derived from natural products.

Against this background, plant-derived natural products have attracted increasing interest in the prevention and management of chronic disorders. Their appeal lies in the fact that, unlike single-target synthetic drugs, they often contain multiple bioactive constituents and may influence several pathological processes simultaneously, which is particularly relevant in multifactorial diseases [9,10]. Traditional Chinese medicine is an important source of such multi-component therapies and has long been employed in the management of headache disorders (including migraine) [11]. In this broad category, medicinal and edible Chinese herbal medicines are of particular interest because they bridge dietary use and therapeutic application, and their long history of human consumption also supports their potential for transformational application. This perspective is particularly important for migraines because the disease is shaped by interconnected metabolic, inflammatory, and neurovascular disturbances, rather than a single pathogenic event.

As a perennial herb in the family Apiaceae, Chaxiong is mainly cultivated in southern China, especially in Jiangxi Province. In traditional Chinese practice, it is regarded as both medicinal and edible. It has long been used to activate qi and improve blood circulation, dispel pathogenic wind, and alleviate pain. In folk use, the rhizome of Chaxiong is often used as a medicinal tea to relieve headaches and migraines [12]. Phytochemical studies have identified a variety of constituents in Chaxiong, including phthalides, organic acids, alkaloids, and volatile components [13,14]. Among related phthalide compounds, butylphthalide has attracted particular attention due to its neuroprotective and cerebrovascular regulatory activities, and has been applied in the clinical treatment of ischemic stroke [15]. Experimental studies have further suggested that Chaxiong has multiple effects on the central and cardiovascular systems and can reduce neuronal damage induced by hypoxia and glucose deprivation [16,17]. Taken together, these findings suggest that Chaxiong may affect migraine-related neurovascular, inflammatory, and metabolic processes. However, despite its long history of traditional use and promising pharmacological properties, the bioactive basis and mechanism underlying its anti-migraine effects remain unclear.

In recent years, advances in high-resolution mass spectrometry and systems pharmacology have enabled the study of complex herbal medicines in a more integrated way. UPLC-Q-TOF-MS/MS can comprehensively characterize the chemical profiles of plant-derived constituents, providing a basis for identifying candidate bioactive compounds [18]. Untargeted metabolomics can capture disease-related metabolic disorders and reveal changes in these disorders after treatment. Network pharmacology further expands the research landscape by connecting candidate constituents with predictive targets and signaling pathways, thereby enabling the elucidation of the role of herbal medicines from a multi-component, multi-target perspective [19]. More importantly, combining metabolomics with network pharmacology allows endogenous metabolic changes to be linked to candidate regulatory nodes. At the same time, molecular validation helps determine whether the predicted pathway-level mechanisms have biological significance in vivo [20]. For migraine, a disease characterized by metabolic, inflammatory, and neurovascular changes, this integration strategy is particularly well suited to mechanistic investigation.

**Figure 1:**
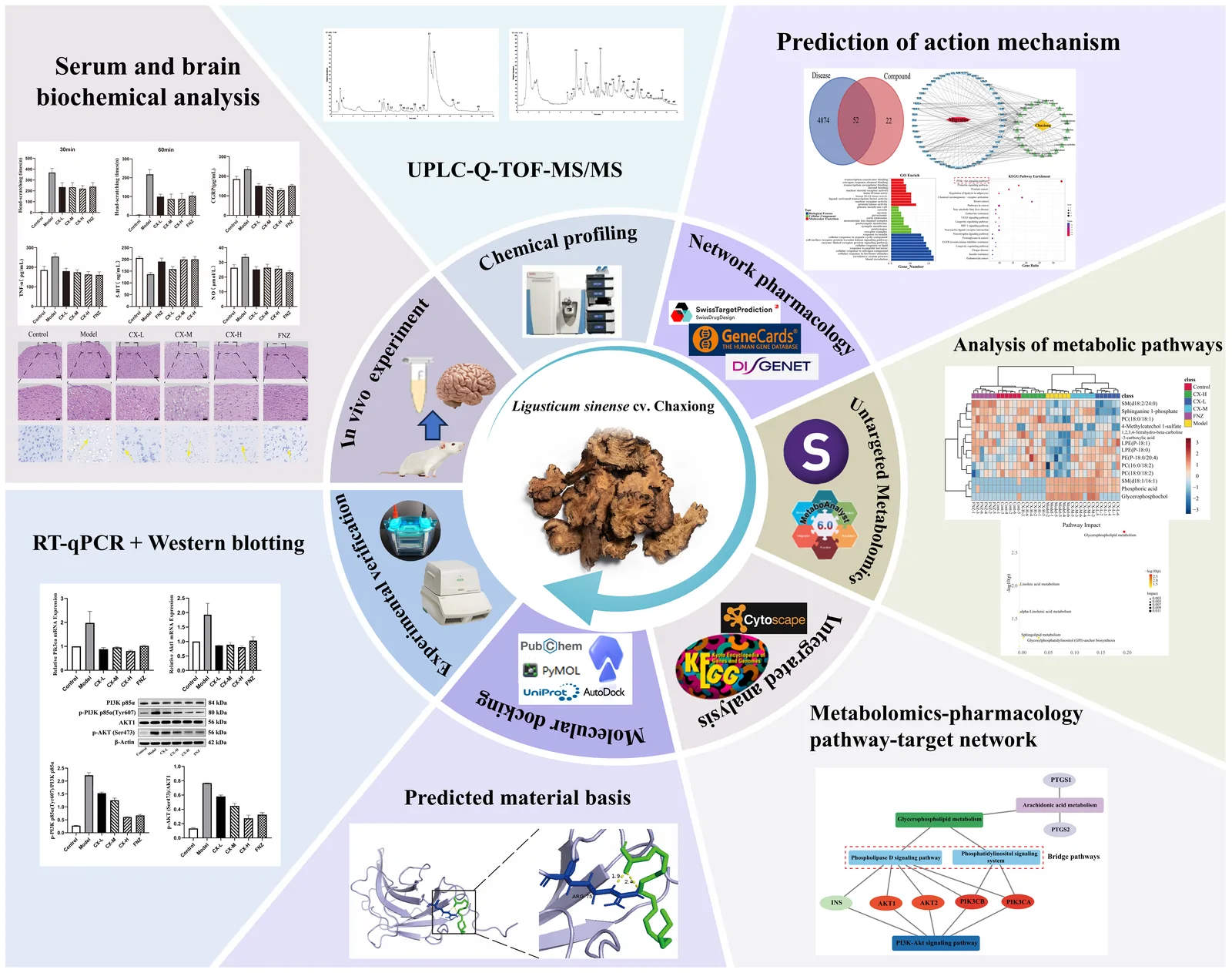
Schematic diagram of this study.

Accordingly, the present study aimed to evaluate the anti-migraine effects of Chaxiong extract and investigate its potential mechanisms in a nitroglycerin-induced migraine model in rats by integrating UPLC-Q-TOF-MS/MS-based chemical profiling, untargeted metabolomics, network pharmacology, molecular docking, and experimental validation (Figure 1). Particular attention was given to the relationship between metabolic disturbance and signaling regulation, especially the potential association between glycerophospholipid metabolism and PI3K/AKT signaling, as indicated by pathway-level integration and shared candidate targets. Through this integrated approach, the study sought to provide robust experimental evidence supporting the traditional use of Chaxiong in migraine relief and to assess its potential as a medicinal and edible Chinese herbal resource for migraine management.

## 2. Results

### 2.1. Results of Behavioral Assessments

**Figure 2:**
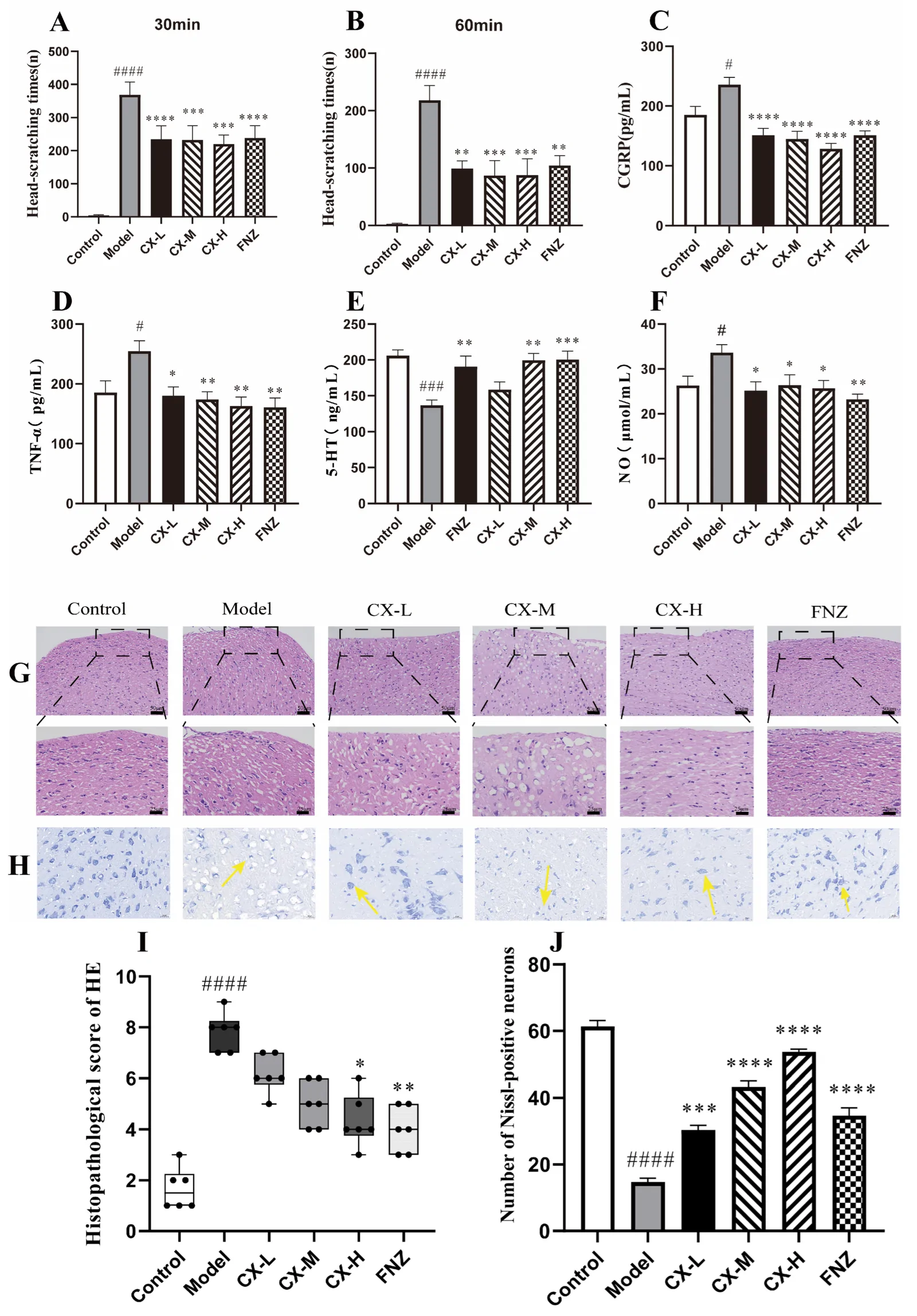
Anti-migraine effects of Chaxiong extract in rats. Behavioral assessments: (**A**) Head-scratching frequency at 0–30 min after NTG injection; (**B**) Head-scratching frequency at 30–60 min after nitroglycerin injection. Biochemical markers: (**C**) CGRP; (**D**) NO; (**E**) 5-HT; and (**F**) TNF-α. Histopathological assessment: (**G**) HE staining; (**H**) Nissl staining. Yellow arrows indicate Nissl-positive neurons; (**I**) semi-quantitative histopathological scores of HE staining; and (**J**) Quantitative analysis of Nissl-positive neurons. Data in (**A**,**B**) are presented as mean ± SEM (*n* = 10 independent animals per group), and data in (**C**–**F**,**J**) are presented as mean ± SEM (*n* = 6 independent animals per group). Histopathological scores in (**I**) are presented as median with interquartile range (IQR), with individual animal values shown (*n* = 6 independent animals per group). Symbols #, ###, and #### indicate *p* < 0.05, *p* < 0.001, and *p* < 0.0001, respectively, versus the control group; *, **, ***, and **** indicate the corresponding significance levels versus the model group.

Head-scratching frequency was analyzed using two-way repeated-measures ANOVA. Significant effects were observed for treatment (F(5,54) = 14.169, *p* < 0.0001, partial η^2^ = 0.567), time (F(1,54) = 59.609, *p* < 0.0001, partial η^2^ = 0.525), and the treatment × time interaction (F(5,54) = 2.454, *p* = 0.0448, partial η^2^ = 0.185). Subsequent Dunnett’s multiple-comparisons tests showed reduced head-scratching frequency in the Chaxiong-treated groups compared with the model group within the predefined observation intervals (Figure 2A,B).

### 2.2. Effects of Chaxiong Extract on Biochemical Markers in Serum and Brain Tissue of Rats

The model group showed marked biochemical disturbances compared with the control group, with significantly increased CGRP, NO, and TNF-α levels and decreased 5-HT levels (*p* < 0.05). Compared with the model group, the CX-L, CX-M, CX-H, and FNZ groups showed significantly increased 5-HT levels and significantly decreased CGRP, NO, and TNF-α levels (*p* < 0.05; Figure 2C–F).

### 2.3. Histopathological Analysis of Brain Tissue in Migraine Rats

HE staining revealed marked pathological alterations in the model group, including vascular dilation and inflammatory cell infiltration, compared with the control group. Semi-quantitative histopathological scores differed significantly among the groups (Kruskal–Wallis H = 29.199, *p* = 2.12 × 10^−5^). Dunn’s multiple-comparisons test showed that the histopathological score was significantly increased in the model group compared with the control group. Chaxiong extract treatment reduced histopathological scores, with a statistically significant reduction observed in the CX-H group, whereas the reductions in the CX-L and CX-M groups did not reach statistical significance. Flunarizine treatment also significantly reduced the histopathological score (Figure 2G,I). Nissl staining showed abundant neurons with clearly visible Nissl bodies in the control group, whereas the model group exhibited neuronal loosening, edema, vacuolar degeneration, and markedly reduced Nissl staining. Quantitative analysis showed a significant decrease in the number of Nissl-positive neurons in the model group compared with the control group, whereas Chaxiong extract treatment significantly increased the number of Nissl-positive neurons (Figure 2H,J).

### 2.4. Chemical Characterization of Chaxiong Extract

**Figure 3:**
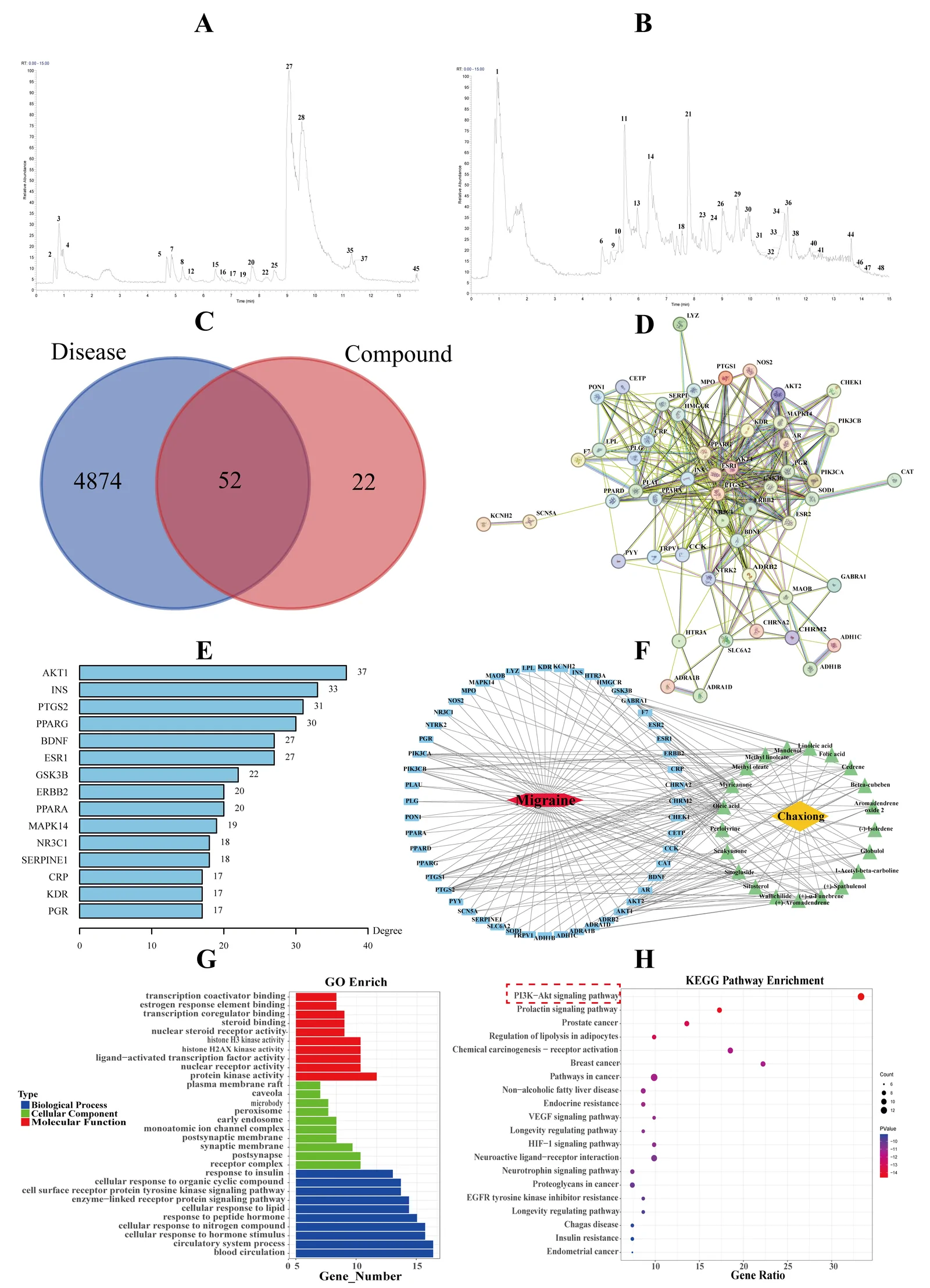
Network pharmacology prediction results. Total ion chromatogram of Chaxiong extract obtained by UPLC-Q-TOF-MS/MS: (**A**) Positive ion mode. (**B**) Negative ion mode. (**C**) Compound–disease target overlap Venn diagram. (**D**) Protein–protein interaction (PPI) network. (**E**) Ranking of the top 15 core targets associated with Chaxiong extract. (**F**) “Drug–Compound–Target–Disease” correlation diagram. (**G**) GO analysis diagram. (**H**) KEGG analysis diagram. The red dashed box in (**H**) highlights the PI3K/AKT signaling pathway.

A total of 48 constituents were annotated based on MS and MS/MS fragmentation patterns through comparison with reference data from public databases, as well as previously published studies (Table S1, Figure 3A,B). Taken together, the chemical profiling results indicate that Chaxiong extract contains multiple constituents with potential relevance to migraine-related inflammatory and neurovascular processes.

### 2.5. Network Pharmacology Analysis of Chaxiong Extract

Based on UPLC-Q-TOF-MS/MS chemical profiling and SwissADME-based screening, 21 candidate constituents were selected from Chaxiong extract (Table S2), and 78 potential targets were predicted using SwissTargetPrediction. In parallel, 4926 migraine-related targets were collected from databases after merging and removing duplicates. Comparison of the two datasets revealed 52 overlapping targets between the predicted targets of Chaxiong extract and migraine-related targets (Table S3), as illustrated in the Venn diagram (Figure 3C). A PPI network constructed from these 52 targets contained 52 nodes and 330 interaction edges (Figure 3D). Degree-based topological analysis using R (version 4.4.2) identified the 15 highest-degree targets (AKT1, INS, PTGS2, PPARG, BDNF, ESR1, GSK3B, ERBB2, PPARA, MAPK14, NR3C1, SERPINE1, CRP, KDR, PGR) potentially relevant to the anti-migraine effects of Chaxiong extract (Figure 3E). To explore this further, a “Drug–Compound–Target–Disease” network was built in Cytoscape with 75 nodes and 208 edges (Figure 3F). Degree-based network analysis ranked Oleic acid, Myricanone, Sitogluside, Linoleic acid, and (+)-α-Funebrene as the most highly connected constituents, indicating that they may represent candidate constituents potentially contributing to the anti-migraine effects of Chaxiong extract. The relative peak abundance of these five representative constituents in the Chaxiong extract was further estimated based on their extracted ion chromatographic peak areas from UPLC-Q-TOF-MS/MS data (Table S4). GO enrichment analysis demonstrated that the common targets were primarily enriched in membrane systems, organelle-associated structures, and synaptic compartments. These targets were involved in molecular functions such as transcriptional regulation and kinase activity, as well as biological processes including signal transduction and hormone responses (Figure 3G). KEGG pathway enrichment further revealed several significantly enriched pathways, including PI3K/AKT signaling, neuroactive ligand–receptor interaction, and prolactin signaling. Among them, the PI3K/AKT pathway appeared to be the most prominent pathway potentially associated with the anti-migraine effects of Chaxiong extract (Figure 3H). Collectively, these observations supported the prioritization of PI3K/AKT signaling as a candidate pathway for further investigation and provided a basis for subsequent pharmacodynamic and metabolomic exploration.

### 2.6. Untargeted Screening of Differential Metabolites

#### 2.6.1. Multivariate Statistical Analysis of Serum Metabolic Profiles Following Chaxiong Extract Treatment

**Figure 4:**
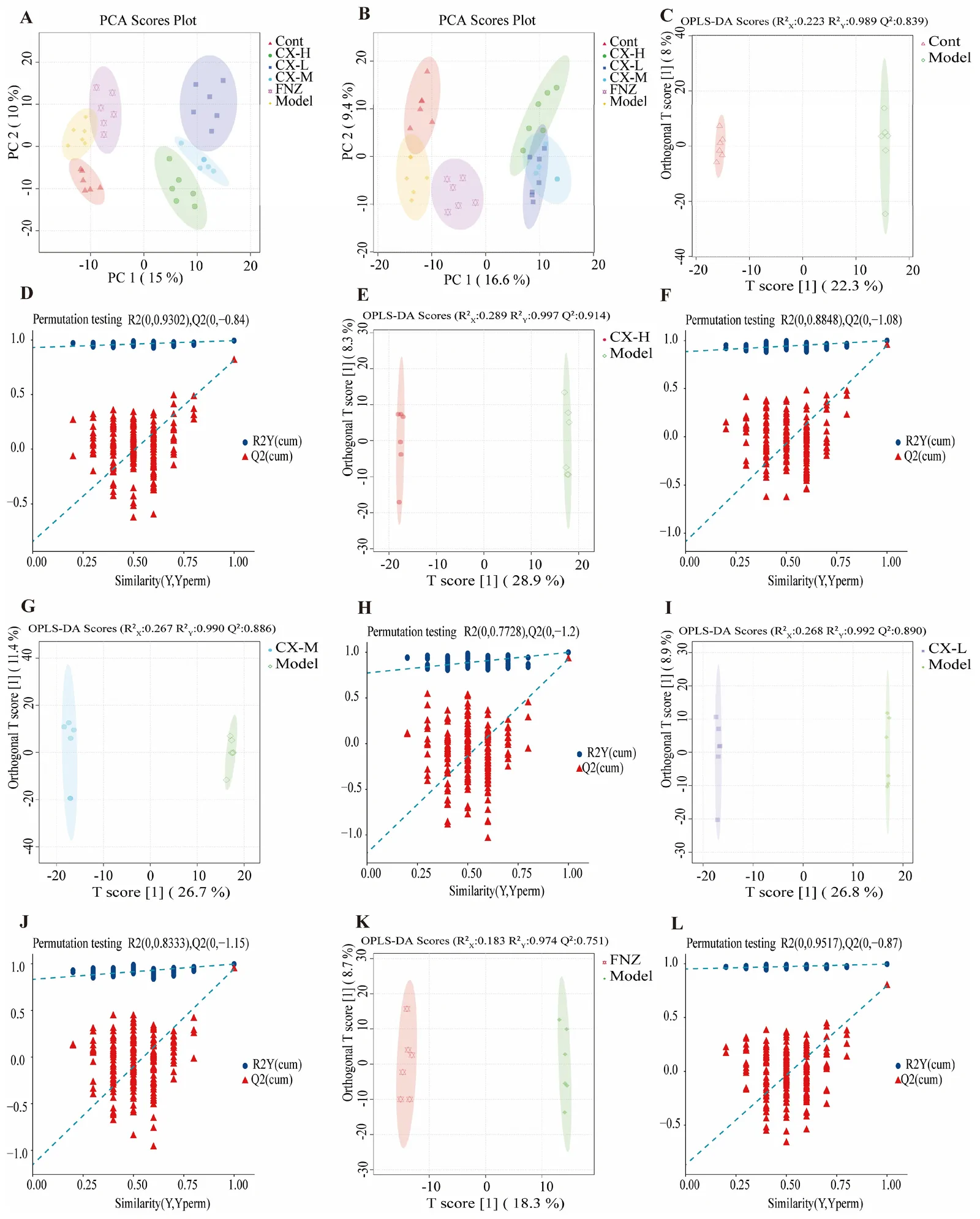
Effects of Chaxiong extract on serum metabolic profiles in rats. PCA score plots: (**A**) Positive ion mode; (**B**) Negative ion mode. (**C**,**E**,**G**,**I**,**K**) OPLS-DA score plots for the indicated group comparisons. (**D**,**F**,**H**,**J**,**L**) OPLS-DA model permutation test.

To ensure the reliability, reproducibility, and robustness of untargeted metabolomics data, three QC strategies were employed in this study prior to downstream statistical analysis. Detailed QC results, including PCA of global samples, correlation analysis of QC samples, and Hotelling’s T^2^ test, are provided in Figures S1–S3. PCA demonstrated a clear separation trend among the groups at the level of global metabolic profiles. Notably, samples from the nitroglycerin-induced migraine model group were distinctly clustered away from those of the control group, reflecting pronounced alterations in systemic metabolic patterns associated with migraine. In contrast, samples from the Chaxiong extract-treated groups were positioned closer to those of the control group, indicating treatment-associated changes in the global metabolic profile (Figure 4A,B).

OPLS-DA analysis demonstrated pronounced differences between the model and control groups, reflecting marked alterations in metabolic profiles induced by nitroglycerin administration. The model showed high goodness-of-fit and predictive ability, with R^2^Y = 0.989 and Q^2^ = 0.938 (Figure 4C). Model validity was further assessed using a 200-permutation test, which indicated no evidence of overfitting (Figure 4D).

Furthermore, OPLS-DA models comparing the model group with the low-, medium-, and high-dose Chaxiong extract groups and the FNZ also showed clear discrimination of their metabolic profiles (Figure 4E–L). CV-ANOVA further confirmed that all OPLS-DA models were statistically significant (Table S5). Collectively, these multivariate analyses suggest that Chaxiong extract treatment modified the nitroglycerin-induced serum metabolic profile.

#### 2.6.2. Analysis of Differential Metabolites and Associated Metabolic Pathways

Hierarchical clustering analysis revealed distinct metabolite expression patterns among the experimental groups and demonstrated the distribution characteristics of the putatively annotated differential metabolites. A total of 13 differential metabolites were putatively annotated, and their detailed information, including MSI levels, VIP values, FC values, Benjamini–Hochberg FDR-adjusted *p*-values, and alteration trends among experimental groups, is summarized in Table S6 and Table 1.

**Figure 5:**
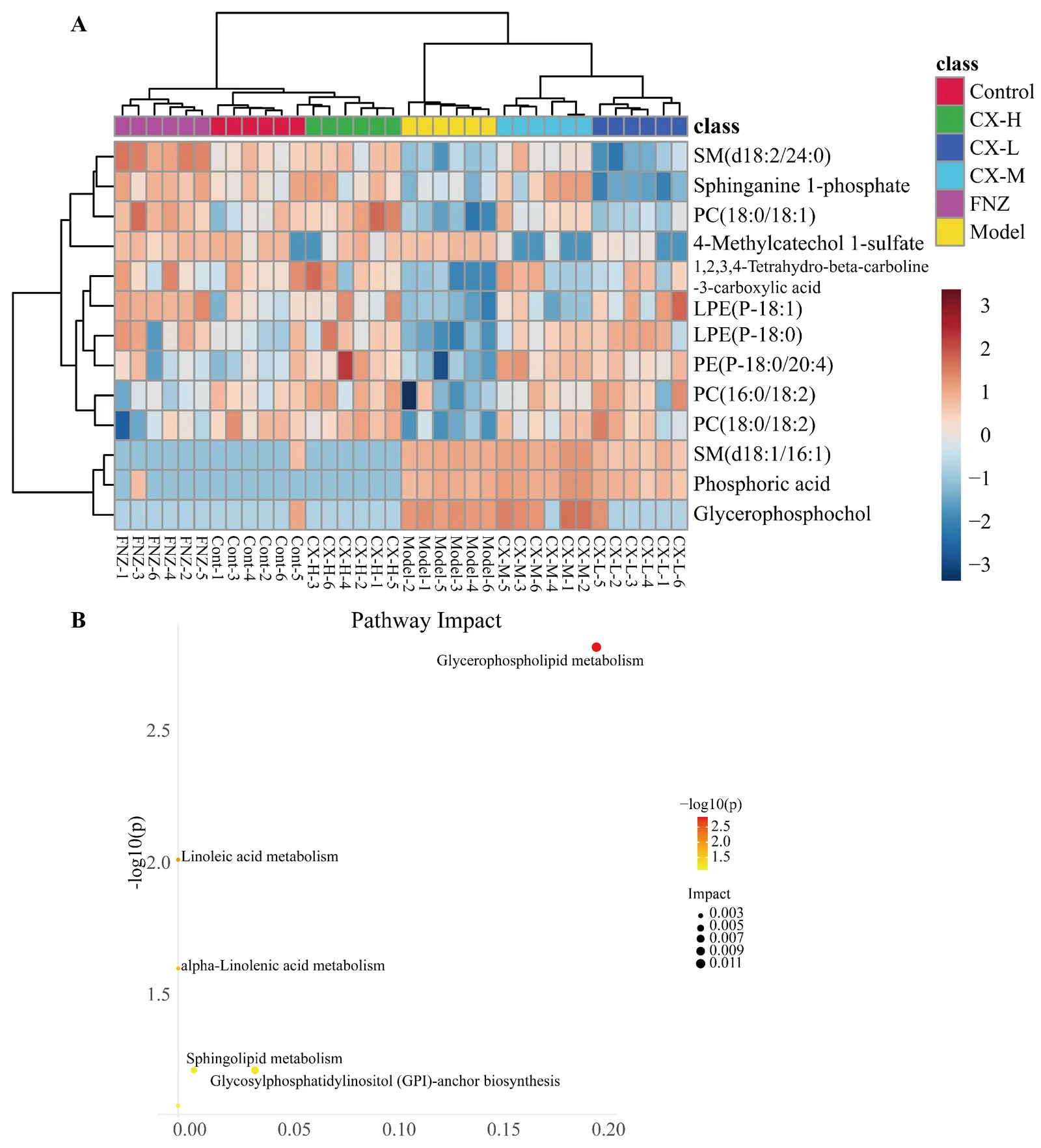
Analysis of differential metabolites and the major metabolic pathways. (**A**) Heat maps of differential metabolites. (**B**) Overview of the major metabolic pathways.

A heatmap was generated to illustrate dynamic changes in serum metabolite levels across all experimental groups (Figure 5A). Relative to the control group, nine metabolites were downregulated and four were upregulated in the model group. Chaxiong extract treatment altered several of these metabolites compared with the model group. Based on Benjamini–Hochberg FDR-adjusted *p*-values, two metabolites showed FDR < 0.01 and an additional seven metabolites showed FDR < 0.05 in the CX-L group. Similarly, two metabolites showed FDR < 0.01 and an additional seven showed FDR < 0.05 in the CX-M group. All 13 metabolites showed significant treatment-associated changes in the CX-H group (FDR < 0.05), including seven with FDR < 0.001, three with 0.001 ≤ FDR < 0.01, and three with 0.01 ≤ FDR < 0.05. The direction of change and statistical significance for each pairwise comparison are summarized in Table 1, with exact FDR-adjusted *p*-values for the additional pairwise comparisons provided in Table S7.

To explore the biological pathways underlying the above metabolic changes, the putatively annotated differential metabolites were imported into the MetaboAnalyst 6.0 platform for enrichment analysis. Based on the screening criteria (impact value > 0.1), one key metabolic pathway was identified: glycerophospholipid metabolism (Table S8, Figure 5B) [21]. Glycerophospholipid metabolism was therefore prioritized for subsequent integration with the network pharmacology results.

### 2.7. Results of the Integrated Analysis of Metabolic Pathways and Network Pharmacology Targets

KEGG enrichment analysis of the untargeted metabolomics data identified glycerophospholipid metabolism as the most significantly enriched pathway among the differential metabolites. Independently, network pharmacology analysis showed that the PI3K/AKT signaling pathway was the most significantly enriched signaling pathway among the 52 overlapping targets. These two independently identified pathways were therefore subjected to further integrative analysis.

**Figure 6:**
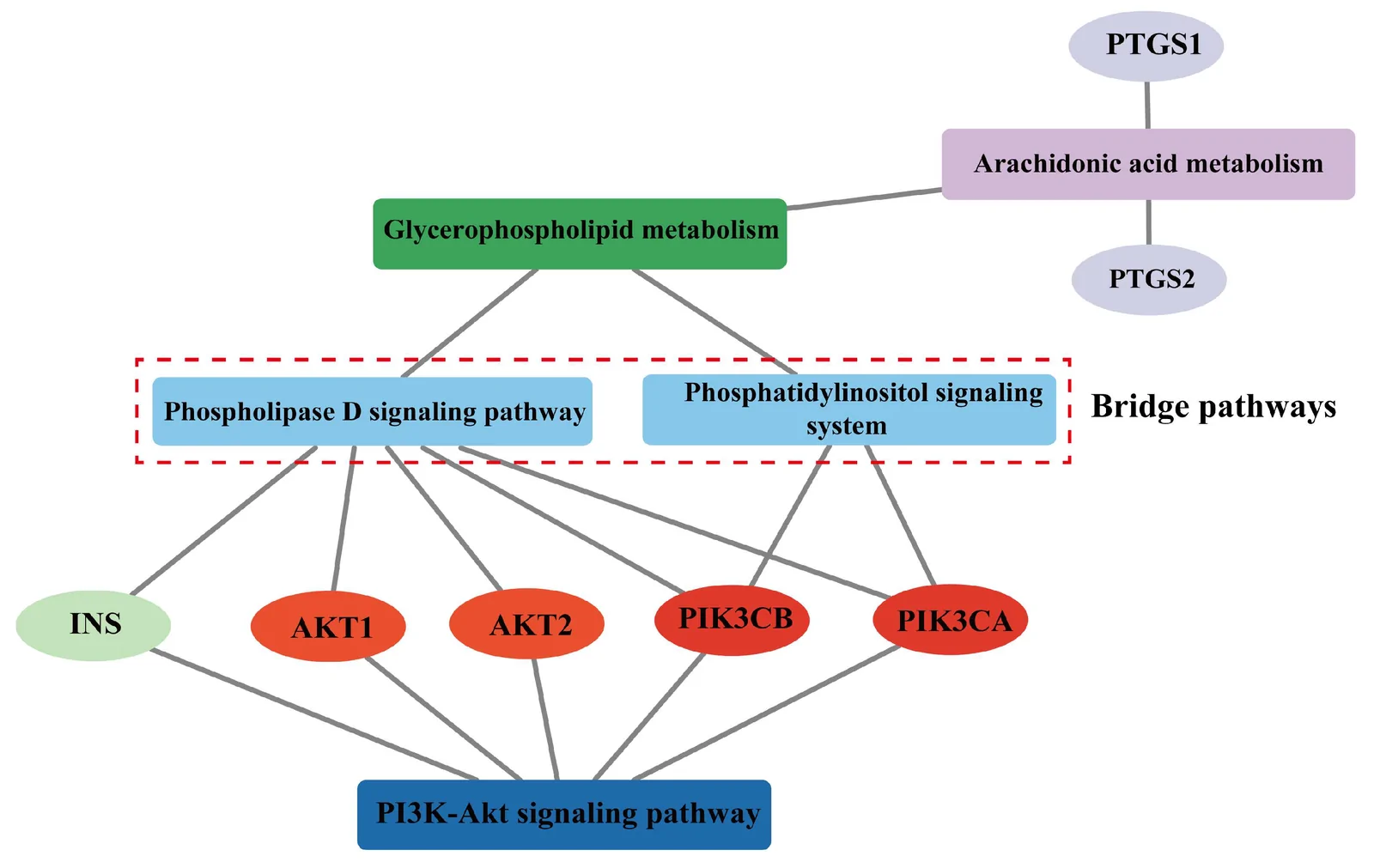
Integrated pathway–target network based on metabolomics and network pharmacology. Glycerophospholipid metabolism and PI3K/AKT signaling, identified as the most significantly enriched pathways by untargeted metabolomics and network pharmacology, respectively, were integrated through KEGG-associated signaling pathways and shared targets. The phospholipase D signaling pathway and phosphatidylinositol signaling system served as bridging signaling pathways, yielding PIK3CA, PIK3CB, AKT1, AKT2, and INS as candidate bridging targets. Arachidonic acid metabolism and its overlapping targets PTGS1 and PTGS2 were included as an auxiliary branch. Edges indicate pathway associations or shared-target relationships rather than experimentally established causal regulation.

Comparison of the phospholipase D signaling pathway with the 52 network pharmacology targets identified five overlapping targets: PIK3CA, PIK3CB, AKT1, AKT2, and INS. The phosphatidylinositol signaling system shared two targets with the network pharmacology dataset, namely PIK3CA and PIK3CB. Further integration with the PI3K/AKT signaling pathway yielded five candidate bridging targets: PIK3CA, PIK3CB, AKT1, AKT2, and INS (Figure 6). Among these, PIK3CA, PIK3CB, AKT1, and AKT2 represented the principal overlapping nodes between glycerophospholipid metabolism-related signaling and the PI3K/AKT pathway. In addition, comparison with the arachidonic acid metabolism pathway identified PTGS1 and PTGS2 as overlapping targets.

### 2.8. Results of Molecular Docking

**Figure 7:**
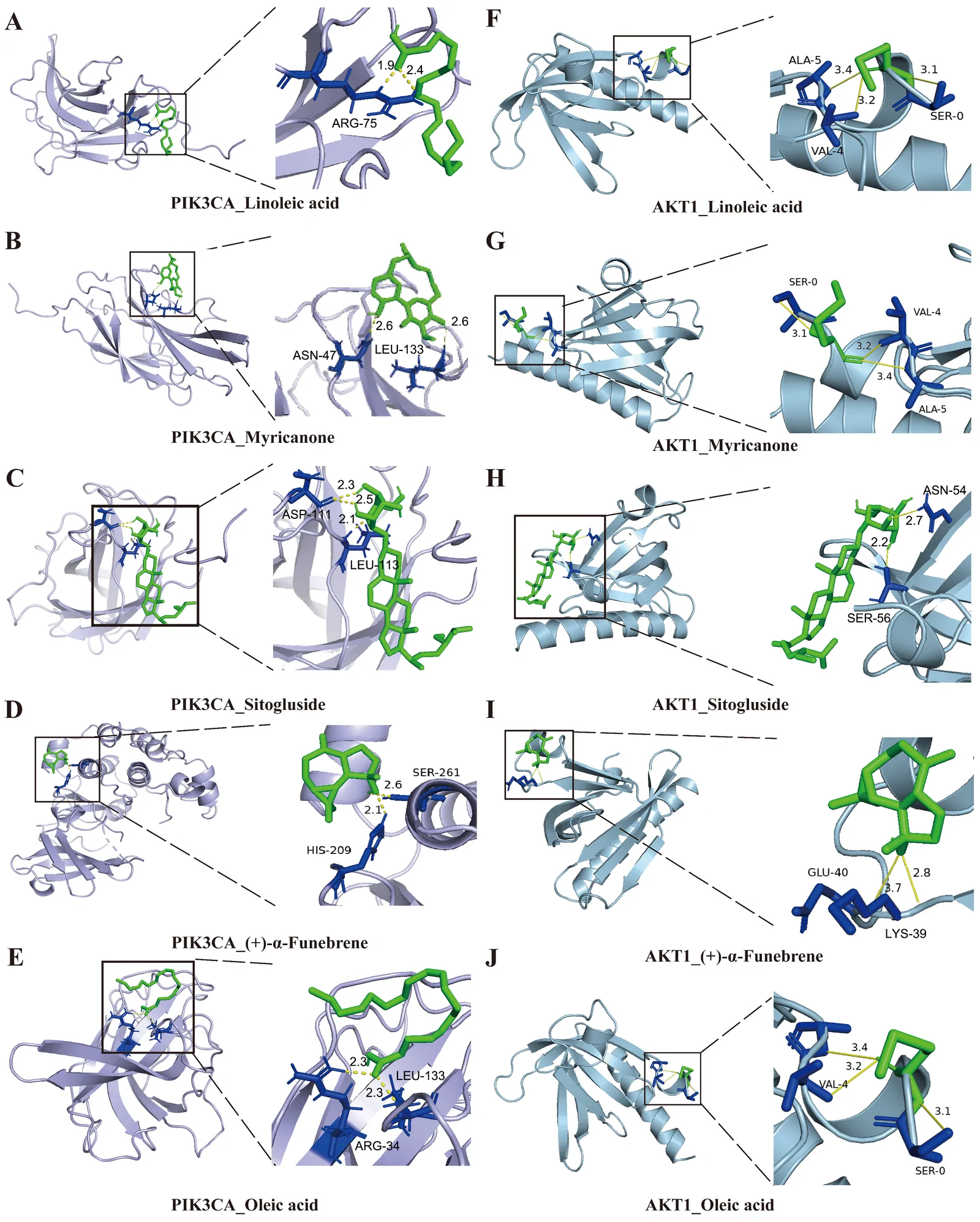
Molecular docking results of representative Chaxiong constituents with PIK3CA and AKT1. (**A**) PIK3CA–Linoleic acid; (**B**) PIK3CA–Myricanone; (**C**) PIK3CA–Sitogluside; (**D**) PIK3CA–(+)-α-Funebrene; (**E**) PIK3CA–Oleic acid; (**F**) AKT1–Linoleic acid; (**G**) AKT1–Myricanone; (**H**) AKT1–Sitogluside; (**I**) AKT1–(+)-α-Funebrene; (**J**) AKT1–Oleic acid. Green sticks represent the docked ligands, blue sticks represent the predicted interacting amino acid residues, and yellow dashed lines indicate hydrogen bonds.

Molecular docking was performed to explore the potential interactions of five representative constituents of Chaxiong extract with PIK3CA and AKT1 (Figure 7). Predicted binding poses were generated for all tested constituent–target pairs using AutoDock Vina, with the corresponding docking scores summarized in Table 2. Among the five tested constituents, Sitogluside showed the most favorable docking scores, with values of −6.8 kcal/mol for PIK3CA and −6.7 kcal/mol for AKT1. The predicted interacting residues for each constituent–target pair are provided in Table S9. These docking results were considered exploratory computational evidence of possible constituent–target interactions rather than confirmation of experimentally validated binding affinity [22].

### 2.9. Pik3ca and Akt1 mRNA Expression Levels Determined by RT-qPCR

**Figure 8:**
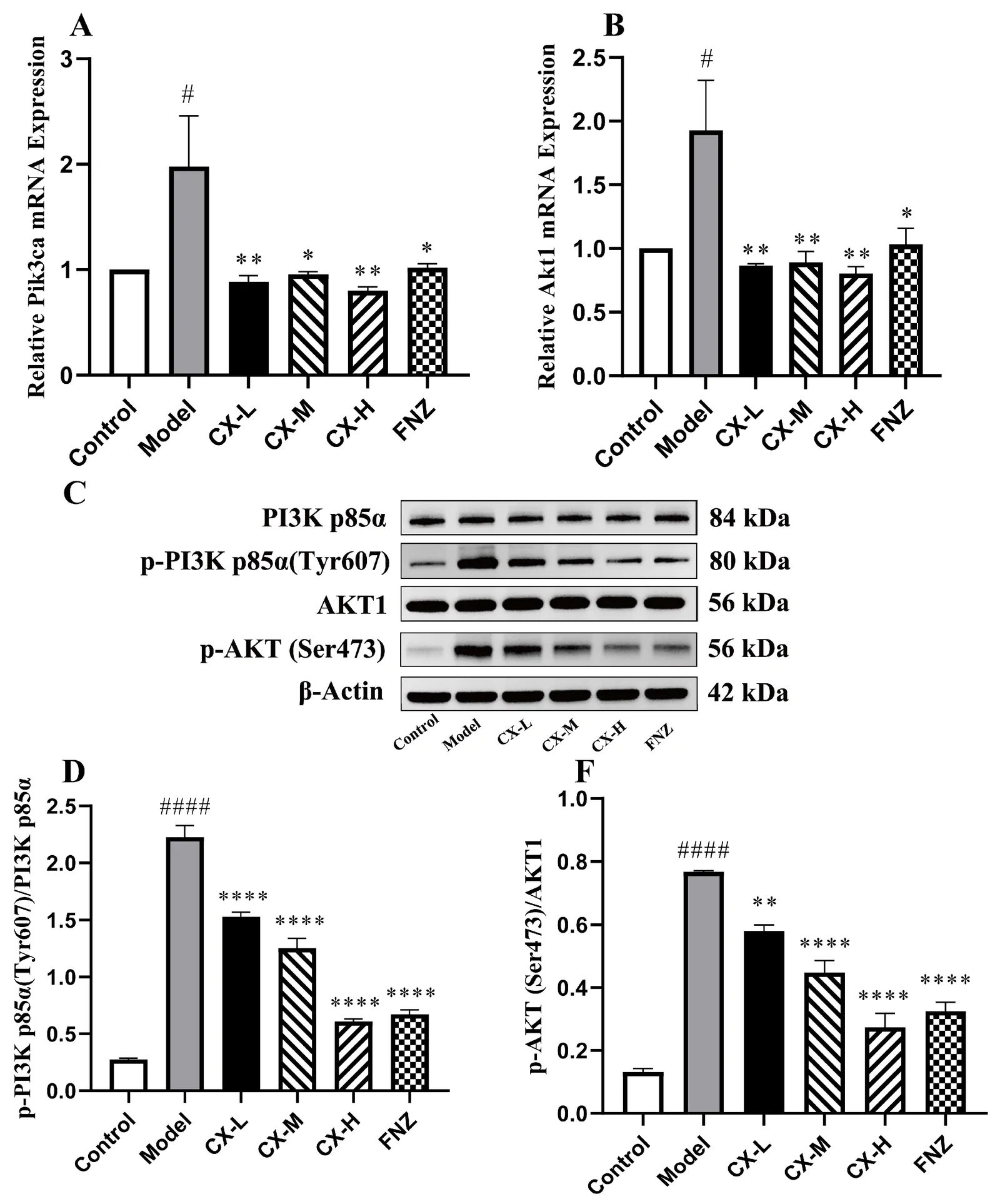
PI3K/AKT signaling-associated molecular changes following Chaxiong extract treatment in migraine rats. (**A**) mRNA level of *Pik3ca*. (**B**) mRNA level of *Akt1*. (**C**–**F**) Effects on the expression of PI3K p85α, AKT1, p-PI3K p85α (Tyr607) and p-AKT (Ser473). Data are presented as the mean ± SEM, with individual data points representing independent animals (n = 3 per group). Compared with the control group, # *p* < 0.05, #### *p* < 0.0001; Compared with the model group, * *p* < 0.05, ** *p* < 0.01, **** *p* < 0.0001).

To examine PI3K/AKT signaling-associated molecular changes following Chaxiong extract treatment, RT-qPCR was used to assess *Pik3ca* and *Akt1* mRNA expression. A marked upregulation of mRNA levels for both genes was detected in the model group relative to control rats (*p* < 0.0001). Chaxiong extract treatment significantly reduced the expression of both genes compared with the model group (*p* < 0.05), with reductions observed across the Chaxiong-treated groups (Figure 8A,B). These findings showed treatment-associated reductions in *Pik3ca* and *Akt1* mRNA expression, two key components associated with the PI3K/AKT signaling pathway.

### 2.10. Changes in PI3K/AKT Signaling-Associated Protein Phosphorylation Following Chaxiong Extract Treatment

Western blotting was used to assess PI3K- and AKT-associated protein phosphorylation in rat brain tissue. Compared with the control group, the model group showed significantly increased p-PI3K p85α (Tyr607)/PI3K p85α and p-AKT (Ser473)/AKT1 ratios (*p* < 0.05). These phosphorylation ratios were significantly reduced following treatment with Chaxiong extract and the FNZ compared with the model group (Figure 8C–F). Individual densitometric quantification of total and phosphorylated PI3K/AKT-related proteins is provided in Figure S4. Taken together, these findings are consistent with reduced PI3K/AKT pathway activation following Chaxiong extract treatment.

## 3. Discussion

Migraine is a complex neurovascular condition involving coordinated interactions among trigeminovascular activation, neurogenic inflammation, altered vascular reactivity, and disturbed neurotransmitter regulation [23]. Once trigeminal afferents are activated, a series of vasoactive and pro-inflammatory mediators are released. CGRP has a central role in this process, as it promotes vasodilation and facilitates nociceptive transmission [24]. Excessive NO production may further exacerbate vascular dilation, central sensitization, and pain signaling, while TNF-α is involved in neurogenic inflammation and tissue damage [25]. In contrast, reduced levels of 5-HT are typically associated with diminished nociceptive regulation during migraine attacks [26]. In the present study, Chaxiong extract significantly alleviated nitroglycerin-induced migraine-like behavior in rats, accompanied by reduced levels of CGRP, NO, and TNF-α, recovery of 5-HT content, and ameliorated brain histopathological alterations. Taken together with the chemical profiling, metabolomic, network pharmacology, molecular docking, and pathway validation results, the findings suggest that the anti-migraine-like effects of Chaxiong extract were accompanied by glycerophospholipid-related metabolic changes and reduced PI3K/AKT-associated signaling. Therefore, the data are consistent with a multi-component pharmacological profile of Chaxiong extract involving concurrent metabolic, inflammatory, and neurovascular changes rather than acting through a single isolated target.

The chemical profile of Chaxiong extract provides a potential chemical basis for the observed pharmacological effects. A total of 48 constituents were annotated by UPLC-Q-TOF-MS/MS, including fatty acids, phenolic compounds, terpenoids, and glycosides. Several of these compounds, such as Oleic acid [27,28], Linoleic acid [29,30], Myricanone, Sitogluside, and (+)-α-Funebrene, have previously been reported to exhibit anti-inflammatory, antioxidant, or neuroprotective activities [31,32,33]. These reported activities are relevant to inflammatory amplification, oxidative stress, and neurovascular dysfunction associated with migraine [34,35]. Although the contribution of each individual constituent remains to be clarified, the coexistence of multiple constituents with potentially relevant activities suggests that Chaxiong extract may contribute to the observed pharmacological effects through coordinated modulation of several pathological processes. This interpretation is consistent with the multicomponent pharmacological characteristics of natural medicinal products rather than a single-compound mechanism [36].

Network pharmacology further indicated that multiple constituents of Chaxiong ex-tract may interact with migraine-related targets. Functional enrichment analysis high-lighted biological processes and pathways related to intracellular signal transduction and neuronal regulation. Among the enriched pathways, PI3K/AKT signaling was particularly prominent, making it a reasonable candidate pathway for further investigation. It should be emphasized, however, that network pharmacology is predictive by nature. Its role in this study was not to establish a mechanism, but to prioritize candidate pathways and guide the integration of the complementary metabolomic and experimental findings.

Untargeted serum metabolomics highlighted glycerophospholipid metabolism as the major metabolic pathway altered in the nitroglycerin-induced migraine-like model and modulated by Chaxiong extract treatment. Several PC-, LPE-, and PE-related metabolites were reduced in the model group, whereas glycerophosphocholine showed the opposite trend. Similar disturbances in circulating phospholipid classes have also been reported in migraine patients. Ren et al. observed decreases in several serum lysophosphatidylethanolamines, including lysoPE 18:1, 18:2, and 22:5, together with reductions in most detected lysophosphatidylcholines [37]. Although the specific molecular species differ from those annotated in the present study, the broadly similar changes at the phospholipid-class level support the relevance of disrupted glycerophospholipid homeostasis to migraine-associated metabolic dysfunction [38]. The reciprocal changes in PC species and glycerophosphocholine are particularly noteworthy because glycerophosphocholine is an important product of phosphatidylcholine catabolism [39], suggesting altered phosphatidylcholine turnover or membrane phospholipid remodeling. In addition, the putatively annotated PE(P-18:0/20:4), assigned as a plasmalogen-type phosphatidylethanolamine, was reduced in the model state and increased following Chaxiong extract treatment. Plasmalogens contribute to neural membrane organization and redox homeostasis, linking their disturbance to oxidative and inflammatory processes relevant to migraine [40]. Thus, the observed glycerophospholipid changes may reflect a broader disturbance of membrane lipid homeostasis rather than isolated alterations in individual metabolites.

The putative annotation of an arachidonate-containing glycerophospholipid pro-vides an additional link between the metabolic findings and inflammatory signaling. PE(P-18:0/20:4) was annotated as containing a 20:4 moiety, while arachidonic acid stored in membrane glycerophospholipids can be mobilized during phospholipid remodeling and subsequently enter eicosanoid biosynthetic pathways. Consistent with this biochemical relationship, arachidonic acid metabolism emerged as an auxiliary branch involving PTGS1 and PTGS2 in the integrated analysis. Cyclooxygenase-dependent lipid signaling is particularly relevant to trigeminovascular inflammation; in primary rat trigeminal ganglion cells, IL-1β has been shown to induce COX-2-dependent PGE_2_ synthesis and subsequently promote CGRP release [41]. These findings provide a plausible biological interface among glycerophospholipid remodeling, inflammatory lipid signaling, and trigeminovascular responses. In the present study, treatment-associated changes in glycerophospholipid-related metabolites occurred alongside reductions in CGRP and TNF-α, supporting an association between lipid metabolic remodeling and improvement of the inflammatory phenotype, rather than establishing a direct arachidonic acid–PTGS–CGRP causal axis.

Beyond this inflammatory lipid branch, pathway-level integration further indicated convergence between glycerophospholipid metabolism-related signaling and PI3K/AKT signaling. The phospholipase D signaling pathway and phosphatidylinositol signaling system provided KEGG-defined bridging pathways, through which PIK3CA, PIK3CB, AKT1, AKT2, and INS were identified as candidate bridging targets, with PIK3CA, PIK3CB, AKT1, and AKT2 representing the principal overlapping nodes. This convergence is biologically plausible because glycerophospholipid remodeling can influence membrane-associated signaling environments, whereas PI3K/AKT signaling is closely linked to lipid-mediated signal transduction and cellular metabolic regulation. Notably, concurrent involvement of glycerophospholipid metabolism and PI3K/AKT signaling has also been reported in another nitroglycerin-induced migraine-like rat model treated with Chuanxiong Qingnao Granules [42], providing independent support for the biological plausibility of the pathway convergence observed here. However, unlike the multi-herb formulation examined in that study, the present work focused specifically on a chemically characterized single botanical extract of Chaxiong. It further provides a Chaxiong-specific chemical and metabolomic profile together with direct examination of Pik3ca/Akt1 expression and PI3K/AKT-associated protein phosphorylation, thereby extending the previous findings to this distinct botanical preparation. Together, these observations suggest that glycerophospholipid remodeling and PI3K/AKT signaling may represent interconnected regulatory layers in the response to Chaxiong extract treatment. Nevertheless, the present evidence supports pathway convergence rather than a linear causal relationship between these processes.

On the basis of the integrated analysis, PIK3CA and AKT1 were selected as representative nodes of the PI3K and AKT arms for further examination. Exploratory molecular docking generated predicted interaction poses between the five representative constituents and both targets, with sitogluside showing the most favorable Vina docking scores among the tested constituents. These computational results provide hypothesis-generating support for possible constituent–target interactions rather than evidence of experimentally confirmed binding. The selected targets were subsequently examined experimentally. RT-qPCR showed that *Pik3ca* and *Akt1* mRNA expression was elevated in the model group and reduced following Chaxiong extract treatment, while Western blotting demonstrated increased p-PI3K p85α (Tyr607)/PI3K p85α and p-AKT (Ser473)/AKT1 ratios in model rats, both of which were reduced following Chaxiong extract treatment. Together, these transcriptional and phosphorylation changes are consistent with reduced PI3K/AKT-associated signaling following Chaxiong extract treatment. It should be noted that the PI3K protein assessed by Western blotting was the p85α regulatory subunit rather than the PIK3CA-encoded p110α catalytic subunit; therefore, the Western blot findings provide pathway-level evidence of treatment-associated changes in PI3K/AKT signaling rather than direct protein-level validation of PIK3CA.

**Figure 9:**
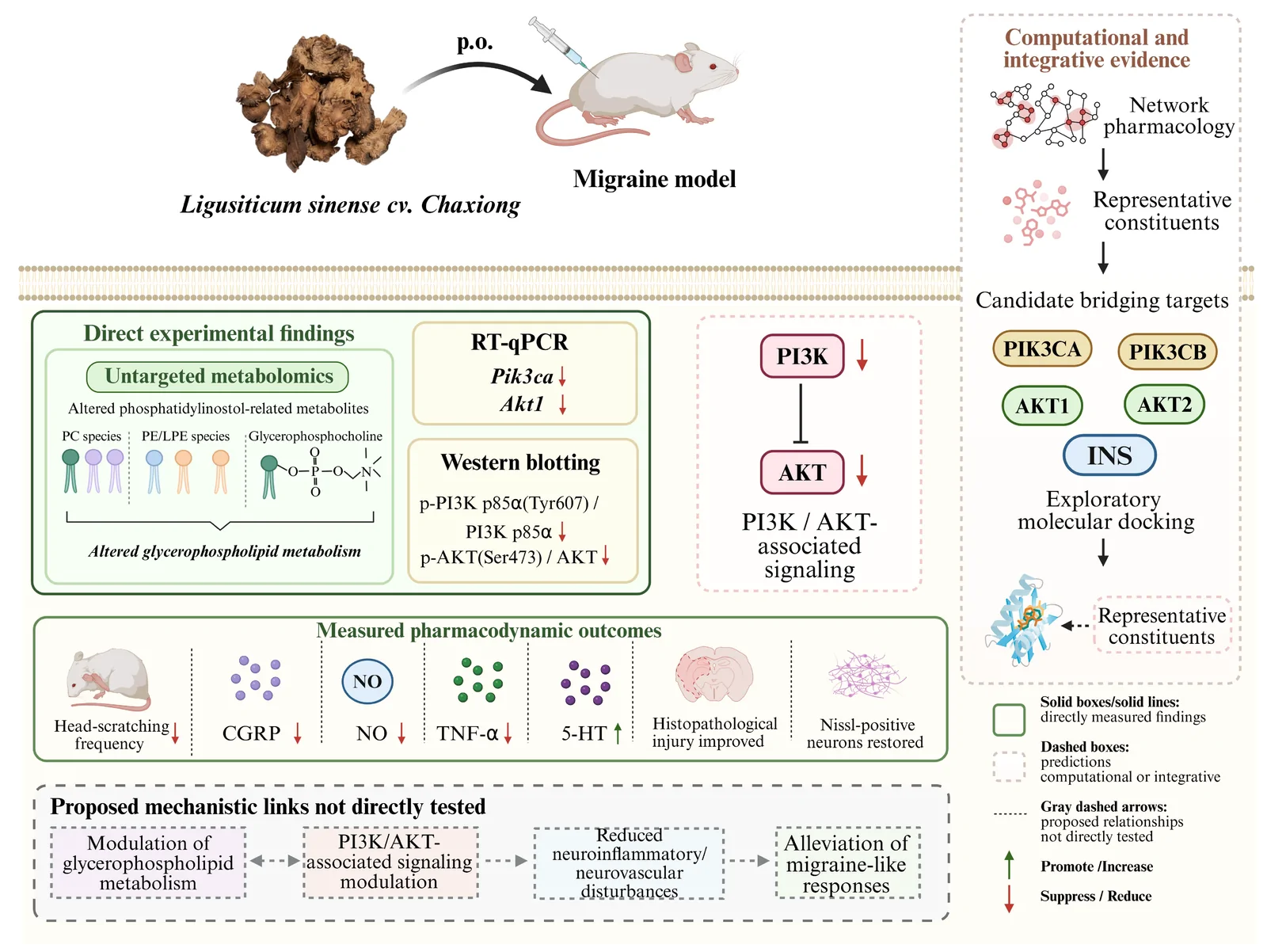
Integrated evidence and proposed working model for the potential anti-migraine effects of Chaxiong extract.

The PI3K/AKT pathway plays broad roles in cell survival, inflammatory regulation, vascular homeostasis, and neuronal function [43]. Dysregulation of these processes is closely associated with migraine pathophysiology, providing a biological context for the increased PI3K/AKT activation observed in the nitroglycerin-induced model. NO may represent one potential link to neurovascular dysfunction, as nitric oxide signaling is an important component of the trigeminovascular system and contributes to vascular responses and nociceptive transmission during migraine [44]. CGRP is another major mediator of trigeminovascular activation, and functional interactions between CGRP and NO signaling have been demonstrated; CGRP can promote NO production, while NO itself contributes to trigeminovascular sensitization and vasodilation [45]. In addition, AKT signaling can interact with proinflammatory cascades, including IKK/NF-κB signaling [46], providing a plausible inflammatory context for the changes in TNF-α observed in the present study. Consistent with these relationships, reduced PI3K/AKT phosphorylation was observed following Chaxiong extract treatment, in parallel with reductions in CGRP, NO, and TNF-α and improvements in migraine-like behavior and brain histopathology (Figure 9). These coordinated changes support an association between lower PI3K/AKT pathway activation and improvement of neuroinflammatory and neurovascular disturbances in the nitroglycerin-induced migraine-like state.

Several limitations should be considered when interpreting the present findings. First, although the metabolomic and network pharmacology findings converged on glycerophospholipid metabolism-related signaling and the PI3K/AKT pathway, the directionality and causal relationship between these processes remain unresolved. Pathway-specific intervention experiments, including PI3K/AKT inhibition, activation, or gene-silencing approaches, were not performed and will be required to determine whether PI3K/AKT signaling is necessary for the pharmacological effects of Chaxiong extract. In addition, the RT-qPCR and Western blot analyses were performed using only three independent animals per group. This limited biological sample size reduces the precision of these molecular estimates; therefore, the corresponding findings should be considered preliminary and require confirmation in studies with larger biological sample sizes. Second, the differential metabolites were putatively annotated using untargeted metabolomics; therefore, targeted lipidomic analysis with authentic standards is needed to confirm individual glycerophospholipid species and quantitatively validate their alterations, particularly the ether/plasmalogen-type lipids. In addition, lipid-remodeling, oxidative-stress, and inflammatory mediators relevant to the proposed biological interpretation, including IL-1β, IL-6, NF-κB, COX-2, and iNOS, were not systematically evaluated. Third, the molecular docking analysis remained exploratory and lacked experimental binding validation, reference-ligand benchmarking, redocking-based RMSD validation, molecular dynamics simulations, and free-energy calculations. The exclusive use of male rats may also limit the generalizability of the findings across sexes. Finally, pharmacokinetic, bioavailability, blood–brain barrier permeability, and quantitative exposure studies of representative Chaxiong constituents were not performed, limiting conclusions regarding their in vivo contribution to the observed effects.

## 4. Materials and Methods

### 4.1. Chemicals and Reagents

Chaxiong was collected from Wuning, Jiangxi, China and authenticated by Professor Zejing Mu (Jiangxi University of Chinese Medicine). A voucher specimen (No. JXUTCM202420019) was deposited in the Herbarium of Jiangxi University of Chinese Medicine. Nitroglycerin injections were sourced from Guangzhou Baiyunshan Mingxing Pharmaceutical Co., Ltd. (Guangzhou, China; batch No. 1230208). Flunarizine hydrochloride was acquired from Renhe Pharmacy Co., Ltd. (Nanchang, China; batch No. H20103601). Commercial assay kits for rat CGRP (Cat. No. ml003082), NO (Cat. No. ml301019), 5-HT (Cat. No. ml003125), and TNF-α (Cat. No. ml002859) were obtained from Shanghai Enzyme-linked Biotechnology Co., Ltd. (Shanghai, China). Furthermore, β-actin (Cat. No.: 66009-1-Ig), AKT1 (Cat. No.: 10176-2-AP), and HRP-Goat anti-Mouse (Cat. No.: SA00001-1) were acquired from Proteintech Group (Wuhan, China). p-AKT (Ser473) (Cat. No. ET1607-73) and PI3K p85α (Cat. No. ET1608-70) antibodies were obtained from HUABIO, while p-PI3K p85α (Tyr607) antibody (Cat. No. AF3241) was obtained from Affinity Biosciences (San Diego, CA, USA).

### 4.2. Preparation and Chemical Characterization of Chaxiong Extract

Chaxiong rhizomes were extracted twice with distilled water at a plant-to-solvent ratio of 1:6 (*w*/*v*) under reflux for 2 h per extraction. The combined aqueous extracts were filtered and concentrated under reduced pressure to prepare the formulations used for animal administration. The extraction yields of the low-, medium-, and high-dose preparations, calculated as the mass of freeze-dried aqueous extract relative to the initial mass of dried Chaxiong rhizome, were 18.02%, 17.75%, and 17.48%, respectively. For chemical profiling, an aliquot of the same aqueous extract was freeze-dried and subjected to analytical sample preparation with pre-chilled methanol/acetonitrile/water (2:2:1, *v*/*v*/*v*) before UPLC-Q-TOF-MS/MS analysis. Detailed extraction and analytical sample-preparation procedures are provided in the Supplementary Materials.

### 4.3. Animal Experiments and Sample Collection

Sixty male Sprague–Dawley rats (SPF, 200 ± 20 g) were obtained from a licensed biotechnology supplier (SCXK (Beijing, China) 2019-0010) and maintained under standard laboratory conditions (22 ± 2 °C, 50% ± 10% humidity, 12 h light/dark cycle) with free access to food and water. All animal experiments were performed in accordance with the guidelines for the care and use of laboratory animals and were approved by the Experimental Animal Ethics Committee of Jiangxi University of Chinese Medicine (Approval No. TEMPOR20230096).

Rats were randomly divided into six groups (*n* = 10 per group): low-, medium-, and high-dose Chaxiong extract groups (CX-L: 0.82 g/kg, equivalent to 148.25 mg dry extract/kg; CX-M: 1.64 g/kg, equivalent to 292.08 mg dry extract/kg; CX-H: 2.46 g/kg, equivalent to 431.41 mg dry extract/kg), a flunarizine hydrochloride group (FNZ: 4.2 mg/kg), a control group, and a model group. No animals were lost or excluded during the study. Animals used for downstream analyses were randomly selected from each group, and all reported *n* values represent independent biological replicates; detailed animal allocation is provided in Table S10.

Treatments were administered once daily by oral gavage for 12 consecutive days. On Day 12, 30 min after the final treatment, rats in all groups except the control group received a single subcutaneous injection of nitroglycerin (10 mg/kg) [47]. Behavioral assessment was performed during the subsequent 0–60 min period. Detailed dosing procedures, including extract concentrations, gavage volume, administration vehicles, dose selection, experimental timing, and blinding procedures are provided in the Supplementary Materials.

At 60 min after nitroglycerin administration on Day 12, immediately following behavioral assessment, rats were anesthetized with 2% sodium pentobarbital (0.2 mL/100 g, i.p.). Blood was collected from the abdominal aorta, centrifuged twice (4500× *g*, 4 °C, 15 min) to obtain serum, and stored at −80 °C. Brain tissue was collected and processed according to the corresponding downstream analyses, as detailed in the relevant sections and Supplementary Materials.

### 4.4. Study on the Interventional Effects of Chaxiong Extract on Migraine

#### 4.4.1. Investigation of Animal Behavior

Utilizing the segmented counting method, we initiated the replication of the nitroglycerin-induced model in rats through subcutaneous injection. We recorded the number of head-scratching episodes occurring within 0 to 60 min post-modeling. The interval from 0 to 30 min was designated as the first time segment, while the period from 30 to 60 min served as the second. The same animals contributed observations to both time intervals. The inhibition rate of head-scratching frequency was calculated using the following formula: (Head-scratching count in the model group − Head-scratching count in the administration group)/Head-scratching count in the model group.

#### 4.4.2. Determination of Blood and Brain Tissue Biochemical Markers

CGRP and NO were measured in serum, whereas 5-HT and TNF-α were measured in brain-tissue homogenate supernatants. Approximately 0.1 g of brain tissue was homogenized on ice with 0.9 mL PBS and centrifuged at 5000× *g* for 15 min at 4 °C. The resulting supernatants were used for 5-HT and TNF-α determination without additional normalization to total protein. All biochemical markers were measured using the corresponding commercial assay kits according to the manufacturers’ protocols. Each biological sample was assayed in triplicate, and the mean value was used for statistical analysis. Analytical ranges, sensitivities, and final reporting units are provided in Table S11.

#### 4.4.3. Brain Histopathology

Brain tissue containing the cerebral cortex was fixed in paraformaldehyde, dehydrated, paraffin-embedded, and sectioned at a thickness of 5 μm. For each animal, three sections were evaluated for HE and Nissl staining, and three non-overlapping microscopic fields per section were randomly selected within the predefined cortical region under the same magnification. HE-stained sections were evaluated according to the predefined semi-quantitative scoring criteria shown in Table S12, with each pathological parameter scored from 0 to 3. Histopathological assessment was performed by an observer blinded to group allocation. Nissl-positive neurons were quantified in the corresponding cortical fields. Measurements obtained from the nine fields were averaged within each animal to generate a single animal-level value.

### 4.5. Network Construction and Analysis

Candidate constituents annotated from Chaxiong extract by UPLC-Q-TOF-MS/MS were further screened using the SwissADME platform (https://www.swissadme.ch/, accessed on 8 September 2026) based on druglikeness and pharmacokinetic properties. The screened constituents were subsequently submitted to the SwissTargetPrediction platform (https://swisstargetprediction.ch/, accessed on 8 September 2026) for potential target prediction. Migraine-associated targets were retrieved from the GeneCards (https://auth.lifemapsc.com/, accessed on 8 September 2026), OMIM (https://www.omim.org/, accessed on 8 September 2026), and DisGeNET (https://disgenet.com/, accessed on 8 September 2026) databases with “migraine” as the search term. These common targets were then imported into the STRING database (version 12.5, https://www.string-db.org/, accessed on 8 September 2026) to construct the protein–protein interaction (PPI) network, with the organism restricted to Homo sapiens and the minimum required interaction score set to 0.400 (medium confidence). A “Drug–Compound–Target–Disease” interaction network was further constructed using Cytoscape (version 3.8.2) to prioritize representative candidate constituents. In addition, functional annotation of the overlapping targets was carried out through Gene Ontology (GO) and Kyoto Encyclopedia of Genes and Genomes (KEGG) enrichment analyses using the Metascape platform (https://metascape.org/, accessed on 8 September 2026).

### 4.6. Untargeted Metabolomic Analysis

After sample preparation, both metabolic and quality control samples were analyzed using a UPLC system (Nexera X2 LC-30AD, Shimadzu, Tokyo, Japan) equipped with an ACQUITY UPLC^®^ HSS T3 column (2.1 mm × 100 mm, 1.8 μm; Waters, Milford, MA, USA), coupled to a Q Exactive Plus mass spectrometer (Thermo Fisher Scientific, Waltham, MA, USA). Raw data were processed in MS-DIAL (version 4.9.221218) for peak extraction, while metabolites were putatively annotated based on accurate mass and tandem mass spectrometry (MS/MS) spectral matching. The resulting data matrix was subjected to principal component analysis (PCA) and orthogonal partial least squares discriminant analysis (OPLS-DA) using SIMCA software (version 14.1). Differential metabolites were subsequently analyzed for metabolic pathway enrichment using MetaboAnalyst (version 6.0, https://www.metaboanalyst.ca/, accessed on 8 September 2026). Detailed experimental procedures are provided in the Supplementary Materials.

### 4.7. Integrated Analysis of Metabolic Pathways and Network Pharmacology Targets

To explore potential links between the untargeted metabolomics and network pharmacology findings, the most significantly enriched pathways identified by the two independent analyses were used for subsequent integration. KEGG enrichment of the differential metabolites identified glycerophospholipid metabolism as the most significantly affected metabolic pathway, whereas network pharmacology analysis showed that the PI3K/AKT signaling pathway was the most significantly enriched signaling pathway among the candidate targets.

Based on pathway relationships annotated in KEGG (https://www.kegg.jp/kegg/, accessed on 8 September 2026), gene sets associated with the phospholipase D signaling pathway (hsa04072) and the phosphatidylinositol signaling system (hsa04070), which provide potential signaling links to glycerophospholipid metabolism, were extracted and compared with the 52 candidate targets obtained from network pharmacology. These overlapping targets were further screened against the PI3K-Akt signaling pathway gene set (hsa04151) to identify candidate targets potentially linking glycerophospholipid metabolism-related signaling with the PI3K/AKT pathway. In parallel, the arachidonic acid metabolism pathway (hsa00590) was examined as an additional branch associated with glycerophospholipid metabolism. The integrated pathway–target relationships were visualized using Cytoscape.

### 4.8. Molecular Docking

Molecular docking was performed as an exploratory, hypothesis-generating analysis to investigate potential interactions between five representative constituents of Chaxiong extract and selected protein targets. Integrated metabolomics and network pharmacology analysis identified PIK3CA, PIK3CB, AKT1, AKT2, and INS as candidate bridging targets associated with glycerophospholipid metabolism-related signaling and the PI3K/AKT pathway. PIK3CA and AKT1 were selected as representative targets of the PI3K and AKT nodes, respectively, for focused molecular docking and were subsequently examined by RT-qPCR.

The structures of the five representative constituents were obtained from the PubChem database (https://pubchem.ncbi.nlm.nih.gov/, accessed on 8 September 2026), and their geometries were energy-minimized using ChemBio3D Ultra software (version 12.0). The stereochemical information contained in the retrieved PubChem structures was retained during ligand preparation. The experimentally determined structures of the PIK3CA C2 domain (PDB ID: 2ENQ, chain A) and AKT1 PH domain (PDB ID: 1H10, chain A) were retrieved from the Protein Data Bank. Receptor structures were prepared using PyMOL (version 3.3.1) by removing water molecules and ligands, where applicable. AutoDockTools (version 1.5.7) was used to add hydrogen atoms, assign charges, and convert the receptor and ligand structures to PDBQT format. No separate pH-dependent protonation-state enumeration was performed.

Molecular docking was performed using AutoDock Vina (version 1.1.2). For PIK3CA, the docking search region was centered at X = 2.217, Y = −0.085, and Z = 0.092 Å, with grid settings of 40 × 60 × 40 points and a spacing of 1.0 Å. For AKT1, the search region was centered at X = 21.875, Y = 14.463, and Z = 7.173 Å, with grid settings of 40 × 40 × 42 points and a spacing of 1.0 Å. The exhaustiveness parameter was left at the AutoDock Vina default value of 8, the energy range was set to 5 kcal/mol, and a maximum of 20 binding modes was generated for each ligand–receptor pair. The top-ranked pose according to the Vina docking score was selected for interaction analysis and visualization using PyMOL (version 3.3.1). The PubChem identifiers of the representative constituents and the predicted interacting residues for each ligand–target pair are provided in Table S9. Docking scores were used only for relative comparison of the predicted poses and were not interpreted as experimentally determined binding affinities.

### 4.9. Determination of Pik3ca and Akt1 mRNA Levels by RT-qPCR

Total RNA was isolated from about 0.1 g of homogenized brain tissue using the Trizol method, and RNA concentration and purity were measured before reverse transcription. One microgram of total RNA was reverse-transcribed to cDNA with a commercial kit, which was then used as a template for PCR amplification of *Pik3ca* and *Akt1*, with *Actb* as the internal reference. The relative expression of target genes was calculated using the 2^−ΔΔCt^ method, and all primer sequences are listed in Table S13.

### 4.10. Western Blotting Analysis of PI3K/AKT Pathway Proteins

Proteins were extracted from rat brain tissue with RIPA lysis buffer, and were subsequently separated by SDS-PAGE using gels selected based on the target proteins’ molecular weights. Subsequently, proteins were transferred to polyvinylidene fluoride (PVDF) membranes. After blocking the membranes with 5% skim milk dissolved in TBST for 1 h at room temperature, membranes were incubated with primary antibodies against β-actin (1:50,000), AKT1 (1:4000), p-AKT(Ser473) (1:5000), PI3K p85α (1:2000), and p-PI3K p85α (Tyr607) (1:2000) overnight at 4 °C. Detailed information on the antibodies used for Western blotting is provided in Table S14. After TBST washes, membranes were incubated with HRP-conjugated secondary antibodies (1:10,000) for 1 h at room temperature. Detection was performed with an enhanced chemiluminescence reagent, and imaging was performed with a UVP chemiluminescence system; grayscale values were analyzed with ImageJ (version 1.46).

### 4.11. Statistical Methods

Statistical analyses were performed using IBM SPSS Statistics (version 22.0) and GraphPad Prism (version 9.0). Normality and homogeneity of variance were assessed using the Shapiro–Wilk and Levene’s tests, respectively. Head-scratching frequency was analyzed using two-way repeated-measures ANOVA, with treatment as the between-subject factor and time as the within-subject factor, followed by Dunnett’s multiple-comparison test. Other continuous outcomes satisfying parametric assumptions were analyzed using one-way ANOVA followed by Dunnett’s multiple-comparison test, with the model group as the reference. Semi-quantitative histopathological scores were analyzed using the Kruskal–Wallis test followed by Dunn’s multiple-comparison test. Parametric data are presented as mean ± SEM, whereas histopathological scores are presented as the median with interquartile range (IQR). The individual animal was considered the statistical unit, and technical replicates were not treated as independent observations. A value of *p* < 0.05 was considered statistically significant. Graphs were generated using GraphPad Prism (version 9.0).

## 5. Conclusions

Chaxiong extract significantly alleviated nitroglycerin-induced migraine-like manifestations in rats. Untargeted metabolomics identified glycerophospholipid metabolism as the principal metabolic pathway affected by treatment, while network pharmacology prioritized PI3K/AKT signaling. Integrative pathway–target analysis identified PIK3CA, PIK3CB, AKT1, AKT2, and INS as candidate bridging targets between glycerophospholipid metabolism-related signaling and the PI3K/AKT pathway. RT-qPCR and Western blotting provided preliminary molecular findings consistent with reduced PI3K/AKT pathway activation following Chaxiong extract treatment. These findings provide supportive evidence for the traditional use of Chaxiong in migraine relief and highlight the potential value of medicinal and edible Chinese herbal resources in the development of multicomponent and multitarget therapeutic strategies for migraine.

## Figures and Tables

**Table 1:** Putatively annotated differential serum metabolites across experimental groups.

Metabolites	Formula	CX-H vs. Model				Model vs. Control	CX-L vs. Model	CX-M vs. Model	FNZ vs. Model
		VIP	FC	FDR	Trend	Trend	Trend	Trend	Trend
SM(d18:2/24:0)	C_47_H_93_N_2_O_6_P	1.94	2.95	5.78 × 10^−6^	↑ ***	↓ *	/	↑ *	↑ **
Sphinganine 1-phosphate	C_18_H_40_NO_5_P	1.62	2.97	0.002	↑ **	↓ *	/	↑ *	↑ **
PC (18:0/18:1)	C_44_H_86_NO_8_P	1.81	2.83	0.0001	↑ ***	↓ **	↑ *	/	↑ *
4-Methylcatechol 1-sulfate	C_7_H_8_O_5_S	1.43	0.01	4.54 × 10^−5^	↓ ***	↑ *	↓ *	↓ *	/
1,2,3,4-Tetrahydro-beta-carboline-3-carboxylic acid	C_12_H_12_N_2_O_2_	1.32	2.21	0.022	↑ *	↓ **	↑ *	↑ *	↑ *
LPE(P-18:1)	C_23_H_46_NO_6_P	1.17	2.40	0.05	↑ *	↓ **	↑ *	↑ *	↑ *
LPE(P-18:0)	C_23_H_48_NO_6_P	1.06	2.66	0.048	↑ *	↓ **	↑ **	↑ **	↑ *
PE(P-18:0/20:4)	C_43_H_78_NO_7_P	1.94	9.13	5.78 × 10^−6^	↑ ***	↓ **	↑ **	↑ **	↑ *
PC (16:0/18:2)	C_42_H_80_NO_8_P	1.50	2.01	0.005	↑ **	↓ **	↑ *	↑ *	↑ *
PC (18:0/18:2)	C_44_H_84_NO_8_P	2.07	4.70	2.53 × 10^−8^	↑ ***	↓ **	↑ *	↑ *	/
SM(d18:1/16:1)	C_39_H_77_N_2_O_6_P	1.47	0.009	0.006	↓ **	↑ ***	/	/	↓ ***
Phosphoric acid	H_3_PO_4_	2.06	0.04	1.02 × 10^−7^	↓ ***	↑ ***	/	/	↓ ***
Glycerophosphocholine	C_8_H_20_NO_6_P	1.43	0.01	4.54 × 10^−5^	↓ ***	↑ *	↓ *	/	↓ *

Note: For Model vs. Control, arrows indicate changes induced by nitroglycerin modeling relative to the control group; for treatment vs. Model comparisons, arrows indicate treatment-associated changes relative to the model group. Significance symbols are based on Benjamini–Hochberg FDR-adjusted *p*-values: * FDR < 0.05, ** FDR < 0.01, and *** FDR < 0.001; ↓: decreased abundance; ↑: increased abundance; /: no significance.

**Table 2:** Molecular docking scores between representative constituents and candidate targets.

Core Targets	Vina Docking Score (kcal/mol)				
	Linoleic Acid	Myricanone	Sitogluside	(+)-α-Funebrene	Oleic Acid
PIK3CA	−3.7	−6.1	−6.8	−5.5	−3.2
AKT1	−3.8	−6.4	−6.7	−5.8	−3.9

## Data Availability

The data supporting the findings of this study are included in the article and Supplementary Materials. The complete raw LC-MS data files, processed feature matrices, and associated metadata generated in the untargeted metabolomics analysis are retained by the authors and are available from the corresponding author upon reasonable request.

## Supplementary Materials

The following supporting information can be downloaded at: https://www.mdpi.com/article/10.3390/ijms27188169/s1. References [48,49,50,51,52,53,54,55,56,57,58,59,60,61,62,63,64,65,66,67,68,69,70,71,72,73,74] are cited in Supplementary Materials.

## Abbreviations

*Ligusticum sinense* Oliv. cv. Chaxiong (Chaxiong); Ultra-Performance Liquid Chromatography—Quadrupole Time-of-Flight Tandem Mass Spectrometry (UPLC-Q-TOF-MS/MS); phosphatidylinositol 3-kinase/protein kinase B (PI3K/AKT); Gene Ontology (GO); protein–protein interaction (PPI); principal component analysis (PCA); Kyoto Encyclopedia of Genes and Genomes (KEGG); 5-hydroxytryptamine (5-HT); Reverse Transcription Quantitative Polymerase Chain Reaction (RT-qPCR); calcitonin gene-related peptide (CGRP); enzyme-linked immunosorbent assay (ELISA); tumor necrosis factor-α (TNF-α); low-dose Chaxiong extract group (CX-L); medium-dose Chaxiong extract group (CX-M); high-dose Chaxiong extract group (CX-H); flunarizine hydrochloride group (FNZ); orthogonal partial least squares-discriminant analysis (OPLS-DA).
