# Supplementary material for: Ligusticum sinense Oliv. cv. Chaxiong Extract Alleviates Nitroglycerin-Induced Migraine-like Responses in Rats: Associations with Glycerophospholipid Metabolism and PI3K/AKT Signaling
Source: Int J Mol Sci. 2026 Sep 14;27(18):8169. doi: 10.3390/ijms27188169 (PMC13607287; doi:10.3390/ijms27188169)
Supplement: Supplementary file 1 [file ijms-27-08169-s001.zip › ijms-4461247-supplementary.pdf]

Ligusticum sinense Oliv. cv. Chaxiong Extract Alleviates Nitroglycerin-Induced Migraine-like Responses in Rats: Associations with Glycerophospholipid Metabolism and PI3K/AKT Signaling

Yu Zhu†, Yanjing Dong†, Shiyi Jiang, Qian Qin, Yu He, Danyang Wu, Shouwen Zhang*, Juan Wei*

The Research Center for Traditional Chinese Medicine Resources and Ethnic Minority, Jiangxi University of Chinese Medicine, 330004 Nanchang, Jiangxi, China

* Corresponding author at: The Research Center for Traditional Chinese Medicine Resources and Ethnic Minority, Jiangxi University of Chinese Medicine, 330004 Nanchang, Jiangxi, China.

E-mail addresses: 19990252@jxutcm.edu.cn (S.Zhang); rm303128@hbmu.edu.cn (J.Wei).

† These authors contributed equally to this work.

Supplementary Materials

1. Chaxiong preparation and extraction

The rhizomes of Chaxiong were collected from Wuning, Jiangxi, China, and were authenticated by Professor Zejing Mu from Jiangxi University of Chinese Medicine as *Ligusticum sinense* Oliv. cv. Chaxiong. A voucher specimen (No. JXUTCM202420019) was deposited in the Herbarium of Jiangxi University of Chinese Medicine. The dried rhizomes were pulverized into powder. For preparation of the low-, medium-, and high-dose formulations, 24.68, 49.36, and 74.04 g of Chaxiong powder, respectively, were extracted twice with distilled water at a plant-to-solvent ratio of 1:6 (w/v) under reflux for 2 h per extraction. After each extraction, the aqueous extract was filtered, and the filtrates from the two extraction cycles were combined. The combined aqueous extracts were concentrated under reduced pressure using a rotary evaporator to a final volume of 300 mL, yielding crude-drug equivalent concentrations of 82.27, 164.55, and 246.80 mg/mL for the low-, medium-, and high-dose preparations, respectively. The extraction yield was determined gravimetrically by freeze-drying an accurately measured aliquot of each concentrated aqueous extract to constant weight and was calculated as the weight of the dried extract relative to the initial weight of the dried Chaxiong rhizome. The extraction yields were 18.02%, 17.75%, and 17.48% for the low-, medium-, and high-dose preparations, respectively. Accordingly, the corresponding dry-extract concentrations were 14.83, 29.21, and 43.14mg/mL, respectively. The concentrated aqueous extracts were stored at 4 °C until animal administration.

For chemical characterization, an aliquot of the same concentrated aqueous

Chaxiong extract used for animal administration was freeze-dried. Approximately 0.5 g of the resulting freeze-dried aqueous extract powder was transferred into a 2 mL centrifuge tube, and 1.0 mL of a pre-chilled methanol/acetonitrile/water (2:2:1, v/v/v) was added. The mixture was subjected to ultrasonic treatment in an ice bath for 1 h and subsequently allowed to stand at −20 °C for 1 h. The sample was then centrifuged at 14,000 × g for 20 min at 4 °C. The resulting supernatant was passed through a 0.22 μm microporous membrane and subsequently subjected to UPLC-Q-TOF-MS/MS analysis.

2. Detailed animal treatment and experimental schedule

Treatments were administered once daily by oral gavage at 10 mL/kg for 12 consecutive days. The CX-L, CX-M, and CX-H groups received aqueous Chaxiong extract at crude-drug-equivalent doses of 0.82, 1.64, and 2.46 g/kg, corresponding to actual dry-extract doses of 148.25, 292.08, and 431.41mg/kg, respectively. The corresponding dry-extract concentrations of the administered formulations were 14.83, 29.21, and 43.14mg/mL, respectively. The Chaxiong formulations were aqueous preparations prepared with distilled water, with no additional organic solvent or suspending agent. The medium dose (1.64 g/kg) represented the rat-equivalent clinical dose, whereas the low and high doses corresponded to 0.5- and 1.5-fold of the equivalent dose, respectively. The control and model groups received an equivalent volume of normal saline. Flunarizine hydrochloride (4.2 mg/kg), whose dose was selected according to a previously published study, was suspended in 0.5% CMC-Na solution.

On Day 12, 30 min after the final oral administration, rats in the model, CX-L,

CX-M, CX-H, and FNZ groups received a single subcutaneous injection of nitroglycerin (10 mg/kg), whereas control rats received an equivalent volume of saline. Behavioral observation was initiated immediately after NTG administration and continued for 60 min, with head-scratching episodes recorded separately during the 0–30 and 30–60 min intervals. At 60 min after NTG administration, immediately after behavioral assessment, rats were anesthetized with 2% sodium pentobarbital (0.2 mL/100 g, i.p.), followed by blood and brain tissue collection.

Treatment administration was not blinded to group allocation, whereas behavioral assessment and histopathological evaluation were performed under blinded conditions. No formal a priori power calculation was performed; the initial group size of 10 animals per group was selected to accommodate the multiple experimental endpoints included in the study. Detailed animal allocation across individual analyses is provided in Table S11.

3. Chemical Profiling of Chaxiong Extract by UPLC-Q-TOF-MS/MS

3.1. Chromatographic conditions

Chromatographic separation of Chaxiong extract was performed on a Shimadzu LC-30A series UHPLC system using an ACQUITY UPLC®HSS T3 (2.1 mm × 100 mm, 1.8 μm) column (Waters Corporation, Milford, MA, USA). Mobile phase: aqueous 0.1 % formic acid (A) - acetonitrile (B).

Flow rate: 0.3 mL/min; injection volume: 2 μL. Gradient elution conditions:0→2 min, 0 % B; 2→6 min, 0 %→48 % B;6→10 min, 48 %→100 % B; 10→12min, 100 % B; 12→13 min, 100 %→48 % B; 13→15 min, 48 %→0 % B. The temperature of the column oven was set at 40 ℃.

3.2. Mass spectrometry parameters

MS analysis of serum metabolites was performed on a Q-TOF/MS (Triple TOF 5600+, AB SCIEX, Framingham, MA, USA) operated both in positive and negative ion modes equipped with a DuoSpray ion source. The optimized parameters were set as follows: spray voltage: 3.8 kV (positive ion) and 3.2 kV (negative ion); capillary temperature: 320 °C; sheath gas flow rate: 30 arb; auxiliary gas flow rate: 5 arb; probe heater temperature: 350 °C. The scanning mode was set to full scan, with an MS scan range of 70–1050 m/z and an MS/MS scan range of 50–1250 m/z.

3.3. Data preprocessing

Analyst software (version 1.6, SCIEX, Framingham, MA, USA) was used for operations. The chemical constituents in Chaxiong extract were annotated based on accurate precursor ion masses and MS/MS fragmentation patterns obtained by UPLC-Q-TOF-MS/MS. The annotation was performed by comparing experimental MS/MS spectra and accurate mass information with publicly available spectral databases (such as MassBank and SpectraBase), MS/MS fragment information from public resources, and previously reported chemical constituents of Chaxiong and related Ligusticum species in the literature. Structural annotation was assigned based on the combined evidence of accurate mass, MS/MS fragmentation patterns, and literature-reported phytochemical information.

4. Untargeted metabolomic analysis

4.1. Metabolic sample collection and processing

Frozen samples were thawed at 4 °C and mixed thoroughly. A 100 μL aliquot was extracted with 400 μL of pre-cooled methanol, followed by ultrasonic extraction in an ice bath for 20 min and protein precipitation at −20 °C for 1 h. After centrifugation (16000 g, 4 °C, 20 min), the supernatant was collected and evaporated to dryness under vacuum. The residue was reconstituted in 100 μL methanol–water (1:1, v/v) and centrifuged (20000 g, 4 °C, 15 min). The supernatant was used for LC–MS analysis. Quality control (QC) samples were prepared by pooling equal volumes of all samples.

4.2. Method conditions

Metabolites were separated on a UHPLC system (Nexera X2 LC-30AD, Shimadzu) equipped with an ACQUITY UPLC®HSS T3 column (2.1 mm × 100 mm, 1.8 μm; Waters). The mobile phases consisted of 0.1% formic acid in water (A) and acetonitrile (B) at a flow rate of 0.3 mL/min with a column temperature of 40 °C. The gradient program was: 0–2 min, 0% B; 2–6 min, 0–48% B; 6–10 min, 48–100% B; 10–12 min, 100% B; 12–12.1 min, 100–0% B; and 12.1–15 min, 0% B. The injection volume was 4 μL.

Mass spectrometric detection was conducted using a Q Exactive Plus mass spectrometer (Thermo Fisher Scientific). It is equipped with a heated electrospray ion source that operates in both positive and negative ion modes. The spray voltage in positive and negative ion modes was set to 3.8 kV and 3.2 kV, respectively. Other parameters include: capillary temperature 320 °C, probe heater temperature 350 °C, sheath gas flow rate 30 ARB, auxiliary gas flow rate 5 ARB, and s-lens RF level 50. Data acquisition uses full-scan mode, with a mass-to-charge ratio (m/z) range of 70 to 1350.

4.3. Data processing and pathway analysis

The raw LC-MS data were processed using MS-DIAL (version 4.90). Metabolites were putatively annotated based on accurate mass information and MS/MS spectral matching against the MS-DIAL public MSP library. Observed retention times were reported for chromatographic reference but were not confirmed against authentic standards. Annotation confidence was evaluated according to the recommendations of the Metabolomics Standards Initiative (MSI), and the corresponding MSI levels are provided in Supplementary Table S6.

The resulting data matrix was subjected to multivariate statistical analysis using SIMCA (version 14.1), including principal component analysis (PCA) and orthogonal partial least squares-discriminant analysis (OPLS-DA). The robustness of OPLS-DA models was assessed by permutation testing (200 permutations), and model significance was further evaluated using CV-ANOVA analysis.

Differential metabolites were screened using the CX-H vs Model comparison as the primary comparison, based on VIP > 1, FC ≥ 2 or ≤ 0.5, and FDR-adjusted p-value < 0.05. For the other pairwise group comparisons, p-values were adjusted separately

using the Benjamini–Hochberg method. Metabolic pathway enrichment analysis was subsequently performed using MetaboAnalyst.

4.4. Quality Control of untargeted metabolomics data

To ensure the reliability, reproducibility, and robustness of untargeted metabolomics data, three complementary QC strategies were implemented in this study prior to downstream statistical analysis:

4.4.1. Unsupervised PCA of global samples

An unsupervised PCA model was constructed based on the normalized metabolic data matrix to visualize global metabolic variations and evaluate the stability of the analytical system. In positive and negative ionization modes, the PCA score plots (Figure S1) showed that pooled QC samples injected at regular intervals throughout the analytical sequence exhibited tight clustering, indicating excellent consistency in retention time, peak intensity, and metabolite detection throughout the entire detection process. Distinct separation trends were observed among the blank group, model group, and Chaxiong intervention groups, suggesting the biological significance of migraine-induced metabolic perturbations and the potential regulatory effects of Chaxiong extract. No obvious outlier samples were detected within the 95% confidence ellipse, confirming the reliability of sample preparation, UPLC-MS data acquisition, and preprocessing workflows.

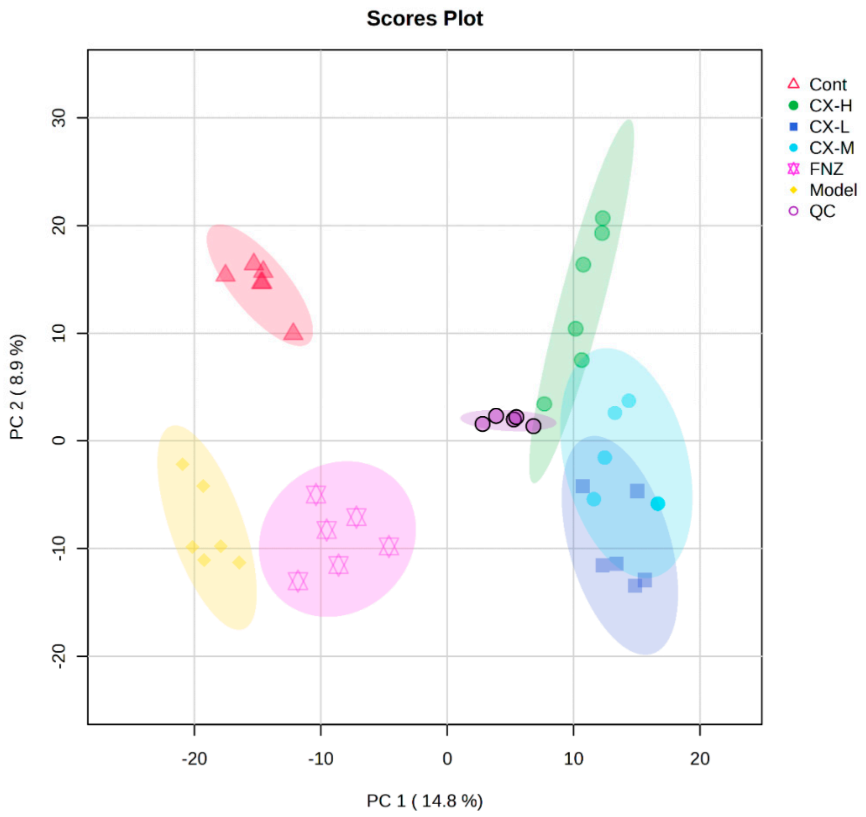

Figure S1. PCA score plot of global samples in combined positive and negative ionization modes

4.4.2. Correlation analysis of QC samples

Technical reproducibility was further validated by calculating pairwise Pearson correlation coefficients between QC samples. As shown in Figure S2, all QC samples exhibited correlation coefficients > 0.9, demonstrating excellent consistency in metabolite profiles across repeated injections. This high level of correlation indicated minimal systematic variation in the analytical platform, including instrument drift, matrix effects, and injection variability, ensuring that the observed metabolic differences between experimental groups were biologically relevant rather than technically induced.

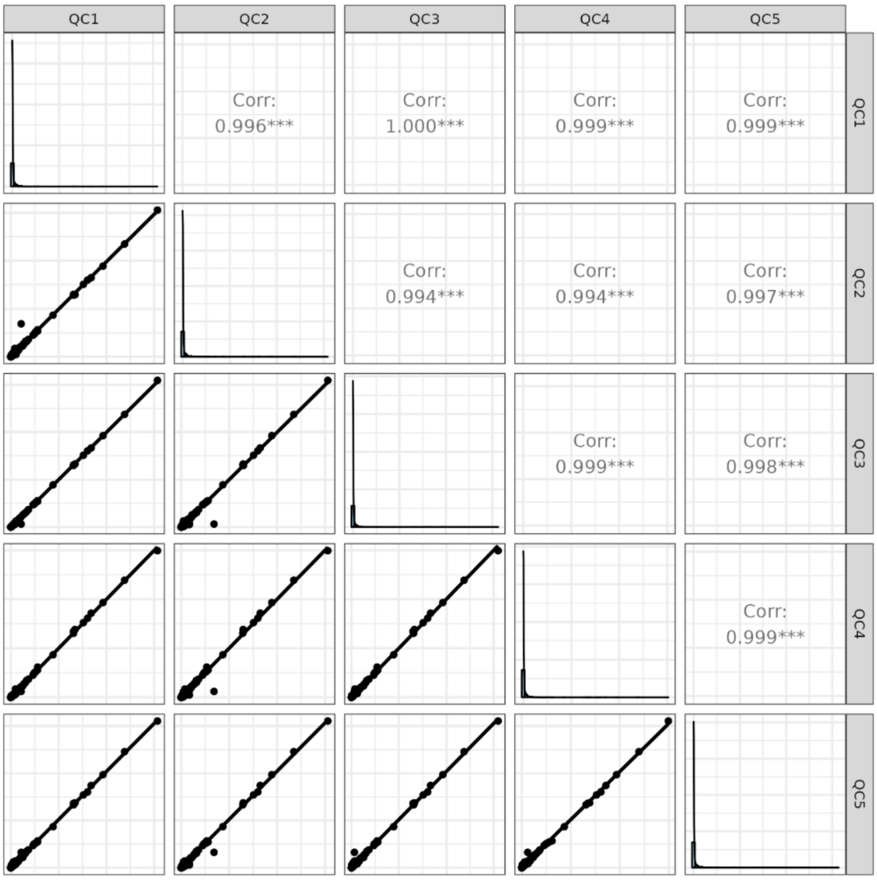

Figure S2. Correlation plot of QC samples in combined positive and negative ionization modes

4.4.3. Hotelling's T^2 test for global samples

Hotelling's T^2 test was employed to identify potential outlier samples with abnormal metabolic profiles, avoiding their interference with the robustness of statistical analysis. With the 95% confidence interval of the Hotelling's T^2 distribution set as the threshold, samples exceeding this interval were marked as outliers. As shown in Figure S3, all experimental samples fell within the 95% confidence ellipse, with samples from the same group colored uniformly.

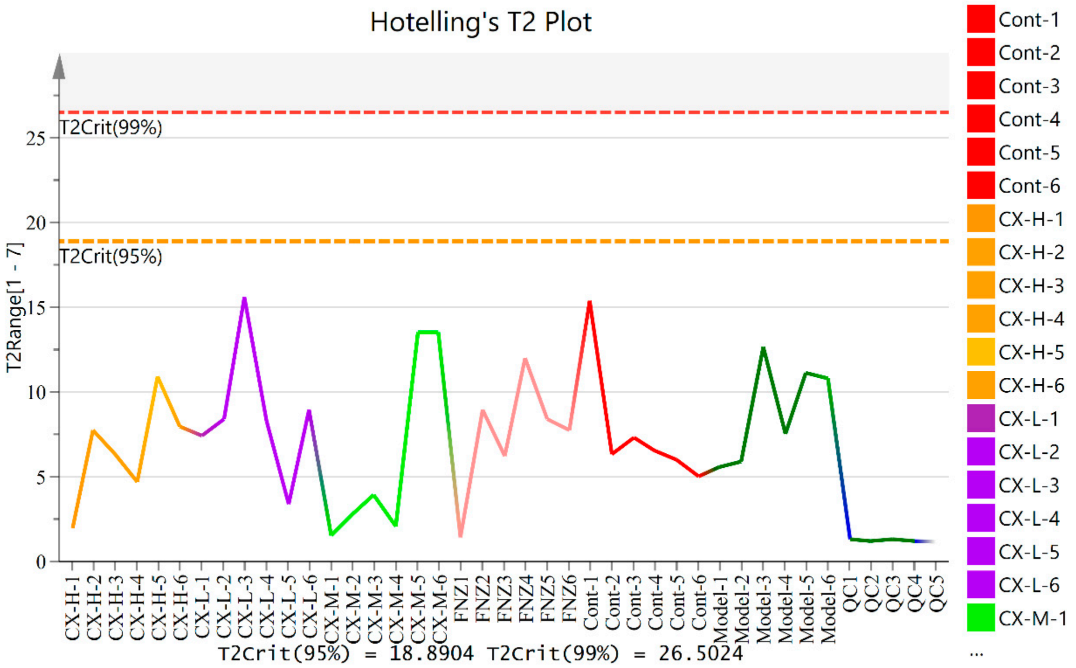

Figure S3. Hotelling's T² test plot of global samples in combined positive and negative ionization modes

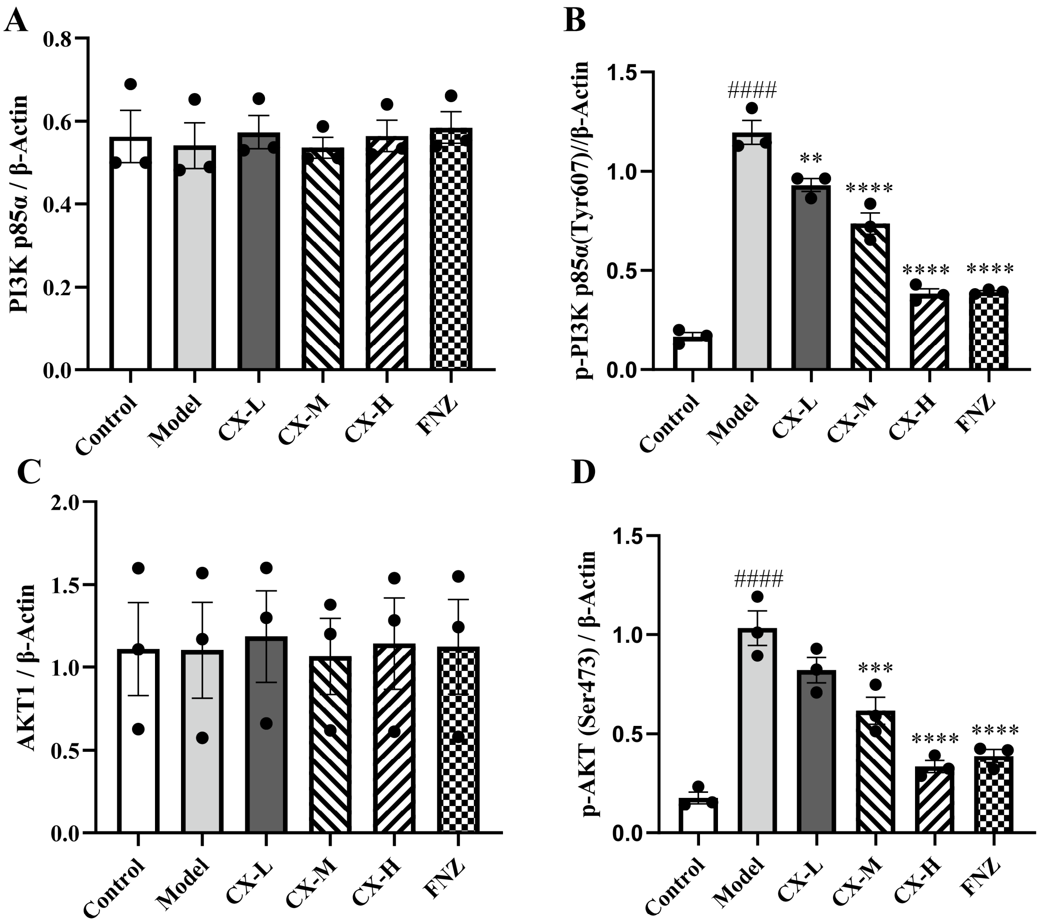

Figure S4. Individual densitometric quantification of total and phosphorylated proteins in the PI3K/AKT signaling pathway. (A) PI3K p85α normalized to β-Actin; (B) p-PI3K p85α (Tyr607) normalized to β-Actin; (C) AKT1 normalized to β-Actin; and (D) p-AKT (Ser473) normalized to β-Actin. Data are presented as the mean ± SEM, with individual data points representing independent animals (n = 3 per group). Compared with the control group, #$P < 0.05$, ##$P < 0.01$, ###$P < 0.001$, and ####$P < 0.0001$; compared with the model group, *$P < 0.05$, **$P < 0.01$, ***$P < 0.001$, and ****$P < 0.0001$.

Table S1. Annotated chemical constituents detected in Chaxiong extract by UPLC-Q-TOF-MS/MS.

Peak No.	RT/min	Annotated constituent	Formula	Adduct	Theoretical m/z	Measured m/z	Error (ppm)	MS^2 fragments	Annotation evidence	Reference
1	0.67	4-Hydroxycinnamic acid	$C_9H_8O_3$	$[M+H]^+$	165.0546	165.0539	-4.24	119.0408,95.0494,77.0381	A+B+D	[1]
2	0.82	Betea-cubebene	$C_{15}H_{24}$	$[M+H]^+$	205.1951	205.1947	-1.95	161.4234,120.9754,105.3426	A+B+D	[2]
3	0.93	Sucrose	$C_{12}H_{22}O_{11}$	$[M-H]^-$	341.1089	341.1092	0.88	179.0621,161.0713,143.0476,113.0587,101.0630	A+B+D	[3]
4	0.96	1-Acetyl-beta-carboline	$C_{13}H_{10}N_2O$	$[M+H]^+$	211.0866	211.0874	3.79	211.0872, 193.0771, 169.0761	A+B+C	[4]
5	4.68	Gallic acid	$C_7H_6O_5$	$[M-H]^-$	169.0143	169.0149	-0.59	150.9601,140.9414,125.0988,97.1437	A+B+D	[5]
6	4.70	Protocatechuic acid	$C_7H_6O_4$	$[M-H]^-$	153.0193	153.0187	-3.92	153.0175,109.0288,91.0195	A+B+D	[6]
7	4.86	(-)-Isoledene	$C_{15}H_{24}$	$[M-H]^-$	203.1805	203.1811	2.95	161.01536,119.5212,105.8280	A+B+C	[7]
8	5.17	Senkyunolide I	$C_{12}H_{16}O_4$	$[M+H]^+$	225.1121	225.1110	-4.89	207.1017,165.0884,143.0824	A+B+D	[8]
9	5.26	Cedrene	$C_{15}H_{24}$	$[M+H]^+$	205.1951	205.1942	-4.39	204.2047,119,7393,93.4526	A+B+D	[9]
10	5.30	Succinic acid	$C_4H_6O_4$	$[M-H]^-$	117.0193	117.0189	-3.42	116.9299,99.9254,73.0283,55.0201	A+B+C	[10]
11	5.42	Sitosterol	$C_{29}H_{50}O$	$[M+H]^+$	415.3934	415.3917	-4.09	119.0857, 116.0530, 79.0218, 57.0706	A+B+C	[11]
12	5.60	Wallichilide	$C_{25}H_{32}O_5$	$[M+H]^+$	413.2323	413.2305	-4.36	413.2335,317.1395,299.1289	A+B+D	[3]
13	5.96	Isochlorogenic acid B	$C_{25}H_{24}O_{12}$	$[M-H]^-$	515.1195	515.1207	2.33	353.0884,335.0770,191. 0560	A+B+D	[12]
14	6.32	(+)-α-Funebrene	$C_{15}H_{24}$	$[M-H]^-$	203.1805	203.1811	2.95	121.2445,119.2703	A+B+C	[13]
15	6.44	Senkyunolide A	$C_{12}H_{16}O_2$	$[M+H]^+$	193.1223	193.1232	4.66	175.1123,147.1167,137.0598,105.0691,91.0547	A+B+D	[3]

16	6.53	3-Butylphthalide	$C_{12}H_{14}O_2$	$[M+H]^+$	191.1067	191.1073	3.14	173.0973,145.1011,135.0442,117.0700,105. 0702,91.0545	A+B+D	[3]
17	6.97	Caffeic acid	$C_9H_8O_4$	$[M-H]^-$	179.0350	179.0356	3.35	135.00577	A+B+D	[3]
18	7.46	Mandenol	$C_{20}H_{36}O_2$	$[M+H]^+$	309.2788	309.2793	1.62	263.3,245.3,213.2	A+B+D	[14]
19	7.61	Sedanolide	$C_{12}H_{18}O_2$	$[M+H]^+$	195.1380	195.1375	-2.56	177.1275,149,1326,125.0598	A+B+D	[15]
20	7.73	N-Butylidenephthalide	$C_{12}H_{12}O_2$	$[M+H]^+$	189.091	189.0915	2.64	171.0829,153.0703,128.0621,115.0547	A+B+D	[3]
21	7.79	Senkyunolide B	$C_{12}H_{12}O_3$	$[M+H]^+$	205.0714	205.0708	-2.93	204.0792,173.0253	A+B+D	[3,14]
22	8.23	(+)-Spathulenol	$C_{15}H_{24}O$	$[M-H]^-$	219.1754	219.1746	-3.65	206.1589,202,1498	A+B+D	[16]
23	8.31	Isochlorogenic acid A	$C_{25}H_{24}O_{12}$	$[M-H]^-$	515.1195	515.1206	2.14	353.0862,191.0570,179. 0349	A+B+D	[12]
24	8.42	Ferulic acid	$C_{10}H_{10}O_4$	$[M-H]^-$	193.0506	193.0512	3.11	178.0952,149.1123,134.0768	A+B+D	[3,8]
25	8.54	(-)-Spathulenol	$C_{15}H_{24}O$	$[M-H]^-$	219.1754	219.1749	-2.28	206.1607,202,1462	A+B+D	[16]
26	9.02	Isochlorogenic acid C	$C_{25}H_{24}O_{12}$	$[M-H]^-$	515.1195	515.1188	-1.36	353.0873,191.0561,179.0536	A+B+D	[12]
27	9.07	Senkyunolide H	$C_{12}H_{16}O_4$	$[M+H]^+$	225.1121	225.1126	2.22	207.0294,191.0192	A+B+D	[17]
28	9.53	Levistolide A	$C_{24}H_{28}O_4$	$[M+H]^+$	381.206	381.2071	2.89	191.1071,173. 0959,163.1118,145.1014	A+B+D	[18]
29	9.57	Chlorogenic acid	$C_{16}H_{18}O_9$	$[M-H]^-$	353.0878	353.0886	2.27	191.0571,173.0456,161.024 3,135.0451	A+B+D	[12]
30	9.95	Salicylic acid	$C_7H_6O_3$	$[M-H]^-$	137.0244	137.0239	-3.65	93.0345,75.0219	A+B+D	[19]
31	10.06	(+)-Aromadendrene	$C_{15}H_{26}O$	$[M-H]^-$	221.1911	221.1903	-3.62	243.1027,227.0714,211.0764,175.0401	A+B+D	[20]
32	10.38	Sitogluside	$C_{35}H_{60}O_6$	$[M-H]^-$	575.4317	575.4330	2.26	411.3632,393.3527	A+B+D	[21]
33	11.16	Coniferyl ferulate	$C_{20}H_{20}O_6$	$[M-H]^-$	355.1187	355.1196	2.53	193.0563	A+B+D	[22]
34	11.24	senkyunolide S	$C_{12}H_{16}O_3$	$[M-H]^-$	207.1027	207.1035	3.86	189.0918,161.0933	A+B+D	[23]
35	11.31	Z-Ligustilide	$C_{12}H_{14}O_2$	$[M-H]^-$	189.1067	189.1074	3.91	191.1076,173.0970,145.1007,117.0701,105.0695,91.0545	A+B+D	[8]

36	11.35	Perlolyrine	$C_{16}H_{12}N_2O$	$[M-H]^-$	247.0877	247.0869	-3.24	264.2863 247.3204,246.1038	A+B+D	[12]
37	11.42	Palmitic acid	$C_{16}H_{32}O_2$	$[M+H]^+$	257.2330	257.2342	4.67	255.2333, 226.1977,195.2303	A+B+D	[12]
38	11.58	Senkyunone	$C_{22}H_{30}O_2$	$[M-H]^-$	325.2173	325.2176	0.92	175.6784,69.0287	A+B+D	[2,23]
39	11.62	Aromadendrene oxide 2	$C_{15}H_{24}O$	$[M-H]^-$	219.1754	219.1761	3.19	201.0193,168.0819	A+B+D	[20]
40	12.15	Globulol	$C_{15}H_{26}O$	$[M-H]^-$	221.1911	221.1922	4.97	177.1274,149.1325,125.0597	A+B+D	[24]
41	12.24	Vanillic acid	$C_8H_8O_4$	$[M-H]^-$	167.0350	167.0342	-4.79	16.0289,123.0439	A+B+D	[25]
42	12.60	Methyl linoleate	$C_{19}H_{34}O_2$	$[M-H]^-$	293.2486	293.2475	-3.75	223.2534,195.0363,167.3965	A+B+D	[26]
43	13.24	Methyl oleate	$C_{19}H_{36}O_2$	$[M-H]^-$	295.2643	295.2654	3.73	189.0910,161.0926,147.0441	A+B+D	[27]
44	13.63	Myricanone	$C_{21}H_{24}O_5$	$[M-H]^-$	355.1551	355.1567	4.51	319.4970,252.0407,176.3155	A+B+D	[28]
45	13.65	Oleic acid	$C_{18}H_{34}O_2$	$[M-H]^-$	281.2486	281.2491	1.78	281.2792,254.1598,239.0669,196.0129	A+B+D	[9,14]
46	13.68	Linoleic acid	$C_{18}H_{32}O_2$	$[M-H]^-$	279.2330	279.2336	2.15	279.2659,261.28767,243.2090,135.1665	A+B+D	[2,6]
47	14.01	Folic acid	$C_{19}H_{19}N_7O$	$[M+H]^+$	362.1724	362.1718	-1.66	295.0933,120.0444,176.0584,106.04226	A+B+D	[2]
48	14.31	Augustic acid	$C_{30}H_{48}O_4$	$[M-H]^-$	471.3480	471.3467	-2.76	379.1708,271.0601	A+B+D	[29]

Note: A represents Accurate mass, B represents MS/MS fragments, C represents spectral database matching, D represents literature report.

Table S2. Candidate constituents from Chaxiong extract selected based on SwissADME evaluation.

number	name
1	oleic acid
2	Myricanone
3	Sitogluside
4	Linoleic acid
5	(+)-α-Funebrene
6	Cedrene
7	Globulol
8	(+)-Aromadendrene
9	Betea-cubebene
10	1-Acetyl-beta-carboline
11	Methyl linoleate
12	(+)-Spathulenol
13	Aromadendrene oxide 2
14	Methyl oleate
15	Mandenol
16	wallichilide
17	Perlolyrine
18	senkyunone
19	sitosterol
20	(-)-Isoledene
21	Folic acid

Table S3. Overlapping targets between Chaxiong extract and migraine.

Number	Name	Number	Name	Number	Name	Number	Name
1	PTGS1	16	KCNH2	31	HTR3A	46	PON1
2	PTGS2	17	ESR1	32	ADRA1D	47	INS
3	PIK3CA	18	AR	33	ADH1B	48	PLG
4	PIK3CB	19	SCN5A	34	PLAU	49	CCK
5	LYZ	20	PPARG	35	SOD1	50	NTRK2
6	SLC6A2	21	F7	36	CAT	51	CETP
7	GABRA1	22	KDR	37	ERBB2	52	PYY
8	TRPV1	23	ADRB2	38	LPL		
9	AKT2	24	ESR2	39	SERPINE1		
10	CHRM2	25	MAPK14	40	BDNF		
11	AKT1	26	GSK3B	41	HMGCR		
12	ADH1C	27	CHEK1	42	MPO		
13	ADRA1B	28	MAOB	43	PPARA		
14	CHRNA2	29	NR3C1	44	PPARD		
15	NOS2	30	PGR	45	CRP		

Table S4. Relative peak abundance of five representative constituents in Chaxiong extract.

Constituent	Peak area	Relative peak abundance (%)
oleic acid	1166490.11	1.45
Myricanone	4116813.74	5.11
Sitogluside	1780947.46	2.21
Linoleic acid	2482105.93	3.08
(+)-α-Funebrene	645788.75	0.80

Note: Relative peak abundance was estimated based on the extracted ion chromatogram (XIC) peak areas obtained from UPLC-Q-TOF-MS/MS analysis. The summed peak area of the 48 annotated constituents was 80,638,590.64. Relative peak abundance (%) was calculated as follows: Relative peak abundance (%) = (individual constituent peak area / total peak area of all annotated constituents) × 100.

Table S5. CV-ANOVA results for OPLS-DA models of metabolomic datasets.

OPLS-DA comparison	F-value	p-value
Control vs Model	12.2813	0.0027
CX-L vs Model	7.4754	0.0114
CX-M vs Model	7.2110	0.0161
CX-H vs Model	10.7679	0.0041
FNZ vs Model	4.65681	0.0377

Table S6. Putatively annotated potential serum biomarkers identified by LC–MS/MS.

Metabolites	RT/min	Adduct	Formula	Measured m/z	Theoretical m/z	Error (ppm)	MS2 fragments	matching score	Identification method	MSI level
SM(d18:2/24:0)	12.59	$[M+Na]^{+-}$	$C_{47}H_{93}N_2O_6P$	835.6623	835.6663	-4.82	776.5937, 652.6006, 262.2530, 206.0553	0.75	MSP	2
Sphinganine 1-phosphate	12.18	$[M+H]^+$	$C_{18}H_{40}NO_5P$	382.2713	382.2717	-1.12	364.2618,266.2840	0.92	MSP	2
PC (18:0/18:1)	12.52	$[M+H]^+$	$C_{44}H_{86}NO_8P$	788.6148	788.6164	-2.00	730.0285,605.9960,580.7907,448.5545		MSP	2
4-Methylcatechol 1-sulfate	6.68	$[M-H]^-$	$C_7H_8O_5S$	203.0005	203.0014	-4.38	185.0060,105.0491	0.92	MSP	2
1,2,3,4-Tetrahydro-beta-carboline-3-carboxylic acid	6.18	$[M+H]^+$	$C_{12}H_{12}N_2O_2$	217.0970	217.0972	-0.69	152.1438, 144.0796, 121.0615	0.92	MSP	2
LPE(P-18:1)	12.19	$[M-H]^-$	$C_{23}H_{46}NO_6P$	462.2980	462.2985	-1.00	214.0486, 196.0379, 152.9961, 140.0118	0.75	MSP	2
LPE(P-18:0)	12.47	$[M-H]^-$	$C_{23}H_{48}NO_6P$	464.31232	464.3141	-3.81	214.0486, 196.0380, 152.9958, 140.0118	0.75	MSP	2
PE(P-18:0/20:4)	12.34	$[M-H]^-$	$C_{43}H_{78}NO_7P$	750.54205	750.5443	-2.93	464.3140, 304.2361, 303.2331	0.75	MSP	2
PC (16:0/18:2)	12.56	$[M+H]^+$	$C_{42}H_{80}NO_8P$	758.5682	758.5694	-1.61	742.5392, 671.4657, 486.2990, 279.2330, 255.2330	0.83	MSP	2

PC (18:0/18:2)	12.54	$[M+H]^+$	$C_{44}H_{84}NO_8P$	786.6025	786.6007	2.33	703.5283,522.3565,224.0693	0.83	MSP	2
SM(d18:1/16:1)	11.80	[M-H]-	$C_{39}H_{77}N_2O_6P$	745.5496	745.5501	-0.66	685.5267, 227.2015, 168.0436	0.75	MSP	2
Phosphoric acid	9.21	$[M+H]^+$	H_3PO_4	98.9846	98.9842	4.04	80.9731,62.9629	0.92	MSP	2
Glycerophosphocholine	1.56	$[M+H]^+$	$C_8H_{20}NO_6P$	258.1104	258.1101	1.16	124.9986, 104.1074	0.83	MSP	2

Note: MSP, mass spectral library format; MSI, Metabolomics Standards Initiative. Metabolites were putatively annotated by matching accurate precursor masses and MS/MS fragmentation spectra against the MS-DIAL public MSP library integrating spectra from MassBank, GNPS, and ReSpect. No authentic standards or confirmatory retention-time matching were used; therefore, these metabolites should be regarded as putative annotations (MSI level 2).

Table S7. Benjamini–Hochberg FDR-adjusted p-values for additional pairwise comparisons of the 13 putatively annotated differential serum metabolites..

Metabolite	Model vs Control FDR	CX-L vs Model FDR	CX-M vs Model FDR	FNZ vs Model FDR
SM(d18:2/24:0)	0.018	/	0.044	0.005
Sphinganine 1-phosphate	0.017	/	0.013	0.007
PC (18:0/18:1)	0.002	0.018	/	0.037
4-Methylcatechol 1-sulfate	0.027	0.046	0.025	/
1,2,3,4-Tetrahydro-beta-carboline-3-carboxylic acid	0.009	0.034	0.037	0.049
LPE(P-18:1)	0.004	0.049	0.024	0.047
LPE(P-18:0)	0.007	0.008	0.003	0.042
PE(P-18:0/20:4)	0.005	0.002	0.004	0.038
PC (16:0/18:2)	0.004	0.047	0.047	0.031
PC (18:0/18:2)	0.006	0.023	0.024	/
SM(d18:1/16:1)	1.72E-05	/	/	2.22E-06
Phosphoric acid	1.83E-05	/	/	8.15E-06
Glycerophosphocholine	0.03	0.041	/	0.032

Table S8. Results of metabolic pathway analysis of serum samples.

Pathway Name	Match Status	p	-log(p)	Holm p	FDR	Impact
Glycerophospholipid metabolism	2/36	0.0014711	2.8324	0.11769	0.11769	0.19969
Linoleic acid metabolism	1/5	0.0093984	2.0269	0.74248	0.37594	0
alpha-Linolenic acid metabolism	1/13	0.024313	1.6142	1	0.64835	0
Sphingolipid metabolism	1/32	0.059134	1.2282	1	0.94614	0.0075
Glycosylphosphatidylinositol (GPI)-anchor biosynthesis	1/32	0.059134	1.2282	1	0.94614	0.03665
Arachidonic acid metabolism	1/44	0.080693	1.0932	1	1	0

Table S9. PubChem identifiers and predicted interacting residues of representative constituents in the molecular docking analysis.

Target	Representative constituent	CID	Interacting residues
PIK3CA	Linoleic acid	5280450	ARG-75
PIK3CA	Myricanone	161748	ASN-47, LEU-133
PIK3CA	Sitogluside	5742590	ASP-111, LEU-113
PIK3CA	(+)-α-Funebrene	9972485	HIS-209, SER-261
PIK3CA	Oleic acid	445639	ARG-34, LEU-133
AKT1	Linoleic acid	5280450	SER-0, VAL-4, ALA-5
AKT1	Myricanone	161748	SER-0, VAL-4, ALA-5
AKT1	Sitogluside	5742590	SER-56, ASN-54
AKT1	(+)-α-Funebrene	9972485	GLU-40, LYS-39
AKT1	Oleic acid	445639	VAL-4, SER-0

Table S10. Allocation of animals across the experimental analyses.

Experimental analysis	Control	Model	CX-L	CX-M	CX-H	FNZ	Statistical unit
Initial allocation	10	10	10	10	10	10	Individual animal
Behavioral assessment	10	10	10	10	10	10	Individual animal
Biochemical measurements	6	6	6	6	6	6	Individual animal
Histopathological analysis	6	6	6	6	6	6	Individual animal
Untargeted metabolomics	6	6	6	6	6	6	Individual animal
RT-qPCR	3	3	3	3	3	3	Individual animal
Western blotting	3	3	3	3	3	3	Individual animal

Note: No animals were lost or excluded during the study. Animals used for individual downstream analyses were randomly selected from the corresponding experimental groups. All reported n values represent independent biological replicates from individual animals; technical replicates, where applicable, were not treated as independent observations.

Table S11. Analytical characteristics of commercial assay kits used for biochemical measurements.

Analyte	Sample matrix	Analytical range	Sensitivity	Final unit
CGRP	Serum	1.25–40 pg/mL	0.1 pg/mL	pg/mL
NO	Serum	0.2–100 μmol/L	0.1 μmol/L	μmol/L
5-HT	Brain homogenate supernatant	0.75–24 ng/mL	0.1 ng/mL	ng/mL
TNF-α	Brain homogenate supernatant	31.25–2000 pg/mL	15.63 pg/mL	pg/mL

Table S12. Criteria for semi-quantitative histopathological scoring of HE-stained brain tissues.

Score	Vascular dilation	Inflammatory infiltration	Neuronal alteration
0	No obvious abnormality	No inflammatory cells	Normal neuronal morphology
1	Mild dilation	Sparse infiltration	Mild structural changes
2	Moderate dilation	Moderate infiltration	Moderate degeneration
3	Severe dilation	Dense infiltration	Marked neuronal injury

Table S13. The primer sequences used in RT-qPCR.

Gene	NCBI RefSeq	Forward Primer Sequences (5′-3′)	Reverse Primer Sequences (5′-3′)	Product Length (bp)	Melting-curve specificity
Actb	NM_031144.3	GCCATGTACGTAGCCATCCA	GAACCGCTCATTGCCGATAG	375	Single peak
Pi3kca	NM_133399.4	AACAAGACGGCTTTGTGACC	AATTCACACACTGGCATGCC	202	Single peak
Akt1	NM_033230.3	TCCCTTCCTTACAGCCCTCAAGT	CACGAGACAGGTGGAAGAAGAGC	104	Single peak

Table S14. Detailed information and validation characteristics of antibodies used for Western blotting.

Target	Molecular identity	Cat. No.	Supplier	Phosphosite	Expected MW	Validation
β-actin	ACTB	66009-1-Ig	Proteintech	/	42 kDa	WB validated, rat
AKT1	AKT1	10176-2-AP	Proteintech	/	56 kDa	KD/KO validated; rat brain WB
p-AKT	phospho-AKT	ET1607-73	HUABIO	Ser473	56 kDa	phosphopeptide-based; treatment-dependent WB; rat-reactive
PI3K p85α	PIK3R1 regulatory subunit	ET1608-70	HUABIO	/	84 kDa	rat brain WB
p-PI3K p85α	PIK3R1 regulatory subunit	AF3241	Affinity	Tyr607	~80 kDa	phosphopeptide-specific; rat-reactive

References

1. Yang, H.; Zeng, L.; Zheng, Z.; Huang, X.; Wu, W.; Deng, F. Analysis of Chemical Constituents of the Ethnic Medicine Prinsepiae Utilis Folium Based on UHPLC-Q/Orbitrap HRMS Technology. *J Chin Med Mater* **2022**, *45*, 2144-2150, doi:10.13863/j.issn1001-4454.2022.09.021.
2. Chowdhary, K.; Kaushik, N. UPLC–MS and Dereplication-Based Identification of Metabolites in Antifungal Extracts of Fungal Endophytes. *Proceedings of the National Academy of Sciences, India Section B: Biological Sciences* **2019**, *89*, 1379-1387.
3. Zheng, P.; Huang, M.; Zhang, J.; Zheng, Y.; Shang, J.; Guo, J.; Liu, M.; Xu, C. Study on the chemical composition of Jiangxi specialty Chinese medicine Chaxiong based on UPLC-Q-TOF-MS/MS. *Nat Prod Res Dev* **2024**, *36*, 52-62, doi:10.16333/j.1001-6880.2024.1.006.
4. 1-acetyl beta carboline. Available online: https://mona.fiehnlab.ucdavis.edu/spectra/browse?query=exists(compound.metaData.name:%27InChIKey%27%20and%20compound.metaData.value:%27KZJWDPNRJALLNS-VJSFXXLFSA-N%27
5. Du, Y.; Yang, H.; Tan, C.; Chen, B.; Luo, l. Rapid Analysis of Chemical Components in Baoxinning Capsules Based on UHPLC-LTQ-Orbitrap-MS/MS Technology. *Chin Pharm J* **2022**, *57*, 1842-1850.
6. Xu, H.; Qiao, X.; Zhang, T.; Yu, G.; Bi, Y.; Fong, X.; Wei, K.; Sun, Y. Analysis of Chemical Components of Miao Medicine Rosae Roxburghii Folium Based onUPLC-Q-Exactive-Orbitrap-MS. *Mod Chin Med* **2022**, *24*, 1850-1860, doi:10.13313/j.issn.1673-4890.20220310003.
7. (-)-Isoledene. Available online: https://spectrabase.com/spectrum/uuHtMOk6wQ
8. Wei, Q. Studies on the chemical constituents of theaerial parts of Ligusticum sinense Oliv.cv.Chaxiong. Peking Union medical colllege, Beijing, 2014.
9. Jia, Z.; Cong, S.; Pan, M.; Liu, L.; Liu, J.; Li, Y.; Xiao, H. Analysis of chemical constituents of Suxiao Jiuxin Pills by UPLC-Q-TOF-MS. *Drug Evaluation Research* **2023**, *46*, 330-341.
10. Succinic acid. Available online: https://massbank.eu/MassBank/search?inchi_key=KDYFGRWQOYBRFD-UHFFFAOYSA-N
11. Sitosterol. Available online: https://mona.fiehnlab.ucdavis.edu/spectra/browse?query=exists(compound.metaData.name:%27InChIKey%27%20and%20compound.metaData.value:%27KZJWDPNRJALLNS-VJSFXXLFSA-N%27)
12. Gong, W. Study on chemistry analysis and planting fertilizer effect of characteristic herb Ligusticum sinense Oliv. cv. "Chaxiong" in Jingxi Province. Jiangxi University of Chinese Medicine, Nanchang, 2023.
13. (+)-α-Funebrene. Available online: https://spectrabase.com/spectrum/F34SeoSplk9
14. Zhang, Q.; Feng, Y.; Li, C.; Li, G.; Liu, X.; Li, H.; Wang, H.; Zhou, L.; Zhang, Y. Rapid identification of phthalides and organic acids in Angelica sinensis based on UPLC-Q-TOF/MS. *China Pharmacy* **2022**, *33*, 579-585+591.
15. Olennikov, D.N.; Fedorov, I.A.; Kashchenko, N.I.; Chirikova, N.K.; Vennos, C. Khellactone Derivatives and Other Phenolics of Phlojodicarpus sibiricus (Apiaceae): HPLC-DAD-ESI-QQQ-MS/MS and HPLC-UV Profile, and Antiobesity Potential of

Dihydrosamidin. *Molecules* **2019**, *24*, doi:10.3390/molecules24122286.
16. He, J.; Tu, H.; Shi, Q.; Zhao, X.; Li, Y.; Guo, M.; Song, C.; Feng, Z. Analysis of Chemical Constituents in Chuanxiong Xiaozhong Zhitong Plaster Based on UPLC-OTOF-MS/MS. *Asia-Pacific Traditional Medicine* **2024**, *20*, 60-64.
17. Yang, J.B.; Wang, A.G.; Wei, Q.; Ren, J.; Ma, S.C.; Su, Y.L. New dimeric phthalides from Ligusticum sinense Oliv cv. Chaxiong. *Journal of Asian Natural Products Research* **2014**, *16*, 747-752, doi:10.1080/10286020.2014.914501.
18. Yang, B. Study on the chemical constituents and biological activity ofLigusticum chuanxiong. Lanzhou University of Technology, Lanzhou, 2022.
19. Serag, A.; Baky, M.H.; Döll, S.; Farag, M.A. UHPLC-MS metabolome based classification of umbelliferous fruit taxa: a prospect for phyto-equivalency of its different accessions and in response to roasting. *RSC Adv* **2019**, *10*, 76-85, doi:10.1039/c9ra07841j.
20. Gao, B.; Yang, J.; Liang, R.; Tian, C.; Chen, J.; Yang, Q.; Zhang, G.; Fan, L.; Wang, Y. Research Progress on Chemical Constituents and Pharmacological Activities of Ligusticum sinense Oliv. Cv. Chaviong. *Mod Chin Med* **2024**, *26*, 1596-1607, doi:10.13313/j.issn.1673-4890.20240318003.
21. Chen, Z.; Dong, Y.; Hu, K.; Zhao, R.; Su, Y.; Liu, J.; Sun, L. Identification of chemical components of Angelicae Sinensis Radix-Chuanxiong Rhizoma herb pairs and absorbed components in serum of rats based on UPLC-Orbitrap-HRMS. *Drug Evaluation Research* **2024**, *47*, 776-791.
22. Emad, A.M.; Rasheed, D.M.; El-Kased, R.F.; El-Kersh, D.M. Antioxidant, Antimicrobial Activities and Characterization of Polyphenol-Enriched Extract of Egyptian Celery (Apium graveolens L., Apiaceae) Aerial Parts via UPLC/ESI/TOF-MS. *Molecules* **2022**, *27*, doi:10.3390/molecules27030698.
23. Wei, Q.; Yang, J.; Ren, J.; Wang, A.; Ji, T.; Su, Y. Bioactive phthalides from Ligusticum sinense Oliv cv. Chaxiong. *Fitoterapia* **2014**, *93*, 226-232, doi:10.1016/j.fitote.2014.01.010.
24. Zhang, K. Chemical Constituents of the Rhizome of Ligusticum chuanxiong Hort. and Their Biological Activities. Shandong University, Jinan, 2021.
25. Li, L. Studies on the chemical constituents of Ligusticum chuanxiong. Tianjin University, Tianjin, 2017.
26. Cai, H.; Qiao, F.; Pei, K.; Duan, Y.; Fan, K.; Liu, X.; Xu, Y.; Wang, X. Investigation on Differences of Chemical Constituentsbetween Sun-Dried and Sulfur-Fumigated Chuanxiong Rhizoma Based on UHPLC-Q-TOF MS and Chemometric Analysis. *Journal of Chinese Mass Spectrometry Society* **2017**, *38*, 127-137.
27. Zhang, C. Study on Separation and Activity of Chemical Componentsin L.sinense Oliv cv. Chaxiong. Xiamen University, 2020.
28. Zhang, Y.; Cao, W.; Zhang, Y.; Zhang, G.; Zhou, Q.; Zhou, Z.; Xu, F. Analysis of Chemical Constituents and Blood-Entering Components of Gardeniae Fructus-Ligusticum sinense Oliv. cv. ChaxiongHerb Pair Based on UPLC-Q-TOF-MS Technology. *J Chin Med Mater* **2022**, *45*, 1638-1643, doi:10.13863/j.issn1001-4454.2022.07.021.
29. Liu, Y.; Meng, X.; Jin, X.; Wang, L.; Liu, S.; Chen, S.; Du, K.; Li, J.; Chang, Y. A comprehensive review of the botany, ethnopharmacology, phytochemistry, pharmacology, quality control and other applications of Ligustici Rhizoma et Radix. *J Ethnopharmacol* **2024**, *323*, 117687, doi:10.1016/j.jep.2023.117687.
